# Water stress limits transpiration and growth of European larch up to the lower subalpine belt in an inner‐alpine dry valley

**DOI:** 10.1111/nph.15348

**Published:** 2018-07-20

**Authors:** Nikolaus Obojes, Armin Meurer, Christian Newesely, Erich Tasser, Walter Oberhuber, Stefan Mayr, Ulrike Tappeiner

**Affiliations:** ^1^ Eurac Research Viale Druso 1 Bolzano 39100 Italy; ^2^ Institute of Forest Botany and Forest Zoology Technische Universität Dresden Dresden 01062 Germany; ^3^ Department of Ecology University of Innsbruck Sternwartestrasse 15 Innsbruck 6020 Austria; ^4^ Department of Botany University of Innsbruck Sternwartestrasse 15 Innsbruck 6020 Austria

**Keywords:** climate change, dendrometer, elevation transect, radial stem variations, sap flow, tree ring width, tree water status

## Abstract

- Climate change will further constrain water availability in dry inner‐alpine environments and affect water relations and growth conditions in mountain forests, including the widespread larch forests.
- To estimate the effects of climate conditions on water balance and growth, variation in sap flow and stem radius of European larch was measured for 3 yr along an elevation transect from 1070 to 2250 m above sea level (asl) in an inner‐alpine dry valley in South Tyrol/Italy. Additionally, long‐term climate–growth relations were derived from tree cores.
- Sap flow and radial growth were reduced in dry periods up to an elevation of 1715 m, leading to maximum annual growth at 2000 m. In a wet year no growth difference between elevations was observed. Long‐term tree ring data showed a positive growth response to precipitation up to 1715 m and to temperature only above 2000 m.
- Our results demonstrate that reduced water availability and higher atmospheric water demand limit larch at low elevation within dry Alpine regions. This indicates a general upward shift of this species’ elevational amplitude upon climate change, and respective negative effects on future silvicultural use and ecosystem services at lower elevations in the European Alps.

## Introduction

Forests are particularly sensitive to climate change because of the long life span of trees, which does not allow for rapid adaptation to environmental change (Lindner *et al*., 2010; APCC, 2014). Rising atmospheric carbon dioxide (CO_2_) concentrations, higher temperatures, and changes in precipitation influence the vegetation period, growth, health, and distribution of trees and increase threats such as pest outbreaks, fires, and droughts (EEA, 2012; IPCC, 2014). In recent decades, average temperatures in the Alps increased more rapidly than the global trend, while no general trend in precipitation was observed (Böhm *et al*., 2001; Beniston, 2005; Rebetez & Reinhard, 2007; Ciccarelli *et al*., 2008). Rising temperatures will extend the growing season, which might have positive effects on tree growth and forest development. However, higher temperatures will intensify drought stress because of a higher evaporative demand and more rain instead of snowfall will reduce the water supply of trees during the early growing season. In central Europe, this will be especially critical in dry inner‐alpine regions (EEA, 2012; Elkin *et al*., 2013; Gobiet *et al*., 2014), and also cause changes in tree species composition (Zweifel *et al*., 2009; Falk *et al*., 2012; Zimmermann *et al*., 2013; Fisher *et al*., 2018).

European larch (*Larix decidua* Mill.) is one of the most important and valuable forest tree species in central Europe and was planted far beyond its natural abundance in the Alps, the Carpathians, and the Sudeten region (Falk *et al*., 2012). Often together with *Pinus cembra* (L.), it forms the forest line community in many regions in the Alps. European larch prefers a rather continental climate with mean annual temperatures up to 13.5°C, dry air with many sunny days, and a minimum yearly precipitation of 450 mm (Wolfslehner *et al*., 2011; Falk *et al*., 2012). It avoids nutrient‐poor sands and waterlogged soils (Kölling & Zimmermann, 2007). Owing to their deciduous needle‐leaves, larches have a shorter growing season to reach similar above‐ground production rates as adjacent evergreen conifers. To this end, they have twofold greater photosynthetic rates enabled by higher nitrogen concentration in the foliage, higher specific leaf area owing to less investment in structural tissues, and a more carbon‐efficient crown shape and canopy structure (Schulze *et al*., 1985; Matyssek, 1986; Gower & Richards, 1990).

Climate change is expected to reduce the suitable habitat for larch plantations in lowland Central Europe (Falk *et al*., 2012). In the Alps, however, larch may benefit from the higher vulnerability of *Picea abies* (L., Karst), the primary species in the upper montane and subalpine zone, to increased temperatures, drought, wind fall, and pests (Schmidt, 2009; Wolfslehner *et al*., 2011; Ganthaler *et al*., 2014; Hartl‐Meier *et al*., 2014). The present study focuses on the potential future limitation and/or potentials of European larch at alpine stands. We monitored growth and water balance of larch trees growing along an elevation gradient between 1060 and 2250 m above sea level (asl) at the long‐term socioecological research (LTSER) site Matsch/Mazia in northern Italy. The site is located in an extremely dry inner‐alpine valley and is thus well suited to study hydraulic limitations (Staffler & Karrer, 2001). The large elevation range of European larch in this region is partially a result of the natural substitution of *Fagus sylvatica*‐dominated communities by larch forests (Staffler & Karrer, 2001; Vacik *et al*., 2010a). Additionally, the anthropogenic promotion of larch in traditional dual‐use sylvipastoral systems (Fontana *et al*., 2014) and reforestation in the late 19^th^ and the 20^th^ century of previously cleared and pastured slopes (Staffler & Karrer, 2005; Vacik *et al*., 2010b) increased the abundance of larch at lower elevations. The variation in climatic conditions along the elevation gradient in an already dry region allows a ‘space‐for‐time’ approach (Becker *et al*., 2007; Körner, 2007b), where comparatively humid and cool conditions at higher elevations represent the present, while dry and warm conditions at lower elevations represent the likely future climate. At multiple sites along the elevation transect, we measured sap flow to monitor transpiration and stem radius fluctuations to determine both tree water status and radial growth. To add a long‐term perspective, we also quantified radial growth trends in relation to climatic conditions during the last decades with stem core samples.

We expected growth limitation by cold temperatures at high elevations but overall increased growth rates in recent decades as a result of increasing temperatures. At medium elevations, we anticipated ideal conditions for larch, with optimal water supply and temperatures allowing high growth rates. At low elevations, the combination of low precipitation and high temperatures should lead to water deficits and respective stomata closure and sap flow reduction during dry periods, and thus to a decrease in growth.

## Materials and Methods

### Study area

This study was conducted at the LTSER platform ‘Matsch/Mazia’ (LTER_EU_IT_097) in the Vinschgau/Val Venosta region in the province of South Tyrol, Italy. Owing to the sheltering effect of surrounding mountains, the area is one of the driest in the Alps. Long‐term radial growth was analyzed at five sites (S1100, S1200, S1700, S2000, S2200) located at elevations between 1060 and 2250 m, while sap‐flow and dendrometer measurements were conducted between 2012 and 2014 at the three mid‐elevation sites. Soil texture was sandy loam at the three lower sites and loam at the two highest site. Soils were rather shallow with depths of *c*. 30 cm, except for S1700 which had slightly deeper soil. While larch is the most abundant tree species above 1500 m, it is rather rare at lower elevations, which were reforested 50–100 yr ago mainly with *Pinus nigra* (Arn.) Trees from all size classes with a diameter at breast height larger than 20 cm were selected at each site (see Table 1).

**Table 1:** Site and tree properties of the five measuring sites

Site	Location	Elevation (m asl) Elevation belt	Aspect	Slope (°)	Ratio of larch (%)	Crown cover (%)	Diameter at breast height (cm)	Tree height (m)	Age^a^ (yr)	Projected crown area (m^2^)	Co‐occurring tree species	Soil type	Soil texture^b^ (sand‐silt‐clay)	Soil depth (cm)
S1100	N 46°40.787 E 10°33.660	1070 High colline	NNW	18	100	70	33.5 ± 1.8	13.5 ± 3.1	120.6 ± 3.6	34.1 ± 14.4	–	Haplic leptosol	Sandy loam 56‐39‐5	25
S1200	N 46°40.663 E 10°34.685	1160 High colline‐montane	WSW	18	50	60	34.4 ± 11.6	18.2 ± 7.2	45.5 ± 3.0	58.5 ± 26.6	*Pinus nigra*	Dystric cambisol	Sandy loam 55‐38‐7	30
S1700	N 46°41.661 E 10°36.772	1715 Low subalpine	ESE	31	100	60	38.8 ± 5.0	19.6 ± 2.3	109.1 ± 4.3	60.2 ± 25.3	–	Haplic leptosol	Sandy loam 76‐16‐8	50
S2000	N 46°41.860 E 10°36.430	1990 Subalpine	SE	27	100	40	42.3 ± 10.0	13.9 ± 1.5	74.2 ± 17.3	58.5 ± 27.7	–	Haplic leptosol	Loam 50‐33‐17	30
S2200	N 46°41.848 E 10°35.854	2250 High subalpine	SO	27	20	30	47.1 ± 17.8	10.3 ± 2.2	92.4 ± 56.3	55.7 ± 32.7	*Pinus cembra*,* Picea abies*,* Pinus mugo*	Haplic leptosol	Loam 46‐37‐17	35

Wood cores were taken at all sites from 10–15 trees; dendrometer (at four trees per site) and sap flow (at six trees per site) measurements were conducted at S1200, S1700 and S2000. Elevation belts are according to Vacik *et al*. (2010b). Diameter at breast height, tree height, age, and projected area are site averages ± SD.

a Cambial age at *c*. 1.3 m above ground.

b According to USDA classification.

### Microclimate

Long‐term average yearly precipitation for Matsch/Mazia (1570 m) is 528 mm, and average yearly temperature is 6.6°C (source: Hydrographic Office, Autonomous Province of South Tyrol). The lower parts of the valley are warmer and drier; at a nearby grassland elevational transect, lapse rates of 0.54 K per 100 m and 12 mm per 100 m for temperature and precipitation, respectively, were measured (Della Chiesa *et al*., 2014). The area has a continental alpine precipitation regime, characterized by low total annual precipitation with convective rainfall events in summer and cold winters with weak Atlantic frontal systems and little precipitation (Hydrographic Office, Autonomous Province of South Tyrol). Frequent dry northerly wind increases the evaporative demand (Vacik *et al*., 2010a).

From spring 2012, air temperature and humidity at a height of 1.5 m and soil water content (SWC using capacitance sensors (ECH2O by ONSET, Bourne, MA, USA) and soil‐specific calibration) at depths of 5, 20 and 50 cm were measured at sites S1200, S1700 and S2000 (sensors and logger by ONSET). Averages of SWC from the tree depths were used for further analysis. To avoid the influence of forest canopy structure, precipitation and solar radiation were measured at a height of 2 m in open grassland sites at each elevation (sensors and logger by ONSET and Campbell Scientific, Logan, UT, USA). Vapor pressure deficit (VPD) was calculated from air temperature and humidity, and potential evapotranspiration (PET) was determined according to Allen *et al*. (1998). Minor data gaps were filled using the R‐package missforest (Stekhoven & Bühlmann, 2012).

Long‐term temperature and precipitation data dating back to 1857 were available from the climate station Marienberg/Monte Maria (1310 m), located 5 km from S1100 (Auer *et al*., 2007). Assuming a linear trend, annual mean temperature increased by 0.11° per decade since the start of measurements in 1857, from an average 5.4°C (1861–1870) to 6.7°C (2001–2010). This rate increased to 0.41° per decade recently (1981–2010). A slight decrease of precipitation by 3.2 mm per decade (1857–2015) was not statistically significant.

### Sap flow

Sap flow was measured at breast height (1.3 m) at six trees per site with tissue heat balance (THB) sensors, described by Čermák *et al*. (2004). We used version P4.2 at S1200 and EMS51 at S1700 and S2000, both by EMS (Brno, Czech Republic). At S1200, at S2000 in 2012, and at S1700 in early 2013, we had two sensors per tree on the north and south sides of the trunk. As a result of technical problems, only one sensor on the north side of the trunk was installed during the rest of the measuring period at S1700 and S2000. In contrast to other methods (e.g. heat dissipation and the heat pulse method), THB sensors are independent of sap wood depth as the electrodes cover the whole sap wood and measurement is integrated over their complete length. Therefore, sap flow rates, which were logged at 10 min intervals, are provided by data loggers per unit trunk circumference (kg h^−1^ cm^−1^) and not per sapwood area (Kučera, 2010). Thus, they were scaled to tree level by multiplication with stem circumference (minus bark and phloem thickness) and integrated to daily sums per tree (l d^−1^; Kučera, 2010).

### Calculation of tree water deficit and radial growth from dendrometer records

Stem circumference variation was measured slightly above breast height (1.8 m) at four trees per site at a resolution of 1 μm and an interval of 10 min with DRL26 band‐type dendrometers by EMS Brno. To reduce the influence of hygroscopic shrinking and swelling of the bark on dendrometer records (DMR) and to ensure close contact with the stem, the dead outermost layers of the bark (periderm) were removed (Zweifel & Hasler, 2001; Gruber *et al*., 2009). For further data processing, stem circumference was transformed to stem radius and hourly averages were calculated.

Irreversible growth‐ and reversible water status‐related stem radius changes were determined according to Zweifel *et al*. (2005), with modifications by Ehrenberger *et al*. (2012), by attributing the measured stem radius variations to changes in stem growth and short‐term daily fluctuations in water status. The growth line (GRO) was defined by a moving maximum of the current and previous dendrometer readings. Daily deviations from this GRO were considered a relative measure of drought‐related tree water deficits (TWD). This assumes that radial stem growth is restricted to periods of stem water saturation, while stem radius variation below a previous stem radius maximum is induced by changing tree water status (Oberhuber *et al*., 2015a; Zweifel, 2016; Zweifel *et al*., 2016).

To simplify the comparison of yearly growth between sites and years, growth curves of each tree were modeled using Gompertz equations (Zeide, 1993; Kahm *et al*., 2010) with the R‐package grofit (Kahm *et al*., 2010); the corresponding parameters maximum amplitude of growth (*A*), maximum growth rate (*μ*), start of growth (*λ*), and inflection point (*I*_p_) were compared between sites and years.

### Dendroecological techniques

To compare intra‐annual growth measured with dendrometers to long‐term growth trends, core samples of 0.5 cm diameter were taken in August 2014 at each site. Two cores of each tree were taken at breast height parallel to the slope to avoid sampling of reaction wood and growth irregularities. In the laboratory, the samples were mounted on a holder and the surface smoothed with a razor blade (Pilcher, 1990). Early‐wood, late‐wood, and total ring widths were measured with a resolution of 1 μm using a digital measuring table (LINTAB 4; Rinntech, Heidelberg, Germany) and the tree‐ring program Tsap‐Win (Rinntech, Heidelberg, Germany). Accurate dating was verified using the program Cofecha (Holmes, 1994), which identifies segments within each tree ring series that may have measurement errors or erroneous cross‐dating. To improve the climate signal, residual chronologies were calculated by removing low‐frequency variability related to tree aging and forest stand development, by fitting a negative exponential curve or a linear regression line to the tree ring series and then applying a cubic smoothing spline with a frequency‐response cutoff set at two‐thirds of the length of each series with the program Arstan (Cook & Holmes, 1984). Dimensionless indices were formed by dividing the observed ring width value by the predicted ring width value. Residual chronologies were derived using ARMA models, with a robust mean value function applied to discount the effects of statistical outliers (Holmes, 1994). Because of its smaller dependence on tree age, basal area increment (BAI, calculated according to Biondi & Qeadan (2008) as ${\text{BAI}}_{t}=\pi R_{t}^{2}-\pi R_{t-1}^{2}$, with *R*_*t*_ the stem radius at the end and *R*_*t*−1_ the stem radius at the beginning of the annual increment) is considered a better indicator for radial growth than tree ring width (Fritts, 1976; Schuster & Oberhuber, 2013a).

### Upscaling

Current and future growth conditions of larch were scaled up to the Alpine and surrounding region based on the yearly climatic water deficit and a minimum yearly average temperature of −2.5°C defining the lower temperature limit of larch (Kölling, 2007). Climatic water deficit was defined as precipitation minus potential evaporation. The latter was calculated based on the Thornthwaite method (Thornthwaite & Mather, 1951; Begueria *et al*., 2017), which was calculated for each transect site (based on the 1981–2010 temperature and precipitation records from the climate station Marienberg/Monte Maria and local lapse rates from Della Chiesa *et al*., 2014) and for each pixel (0.8 × 0.8 km) of the maps. Climate warming was simulated based on a scenario for the Alpine region with an average temperature increase of 3.2°C and a precipitation reduction by 14.5% (expected for the end of the 21^st^ century; Gobiet *et al*., 2014).

### Statistical analysis

We used daily values for intra‐annual correlation analysis to reduce the size of the dataset and to avoid the problem of stem capacitance affecting correlations with climate conditions (Oren *et al*., 1998; Ewers *et al*., 1999; Luis *et al*., 2005; Matheny *et al*., 2015). Relations of site means of daily sums of sap flow, daily radius change (DRC), and daily maximum TWD vs climate parameters were calculated using general additive mixed models (GAMM), employing the R packages mgcw (Wood, 2006) and nlme (Pinheiro *et al*., 2017). We followed the protocol by Zuur *et al*. (2009) to account for autocorrelation of the data, heterogeneity of residuals, and nonlinear effects between explanatory and response variables. As explanatory variables, we used the daily sum of PET summarizing air temperature and humidity, radiation and wind speed and representing water demand as well as daily means of SWC and/or daily sums of precipitation describing water availability. In a second step we used VPD and radiation as explanatory variables instead of PET in addition to precipitation and SWC. Possible interactions with measuring sites as well as lag effects (1 and 3 d) in the relation between the explanatory and the response variable were tested and accounted for in case they improved the model. Sap flow was correlated with climatic conditions for the period when neither development nor coloring and cessation of larch needles reduced daily maxima at any site (Julian day (DOY) 150–250) (Zimmermann *et al*., 2000; Luis *et al*., 2005; Wieser & Leo, 2012). According to Deslauriers *et al*. (2007) we used only data from the main period of stem growth to estimate the effect of climatic conditions on daily growth rates. As main growth periods, we defined periods with continuously increasing DMR, high daily growth rates, and a minimum daily TWD close to 0, which differed between years and sites (Table 2). To estimate the influence of water demand (PET) and water supply (SWC, precipitation) on tree water status‐related stem radius variations, we also set up models for DRC and TWD for late summer (DOY 225–275), when yearly stem growth was mostly completed.

**Table 2:** Main growth periods (defined as periods with continuously increasing dendrometer records (DMR)/potential growth (GRO), high daily growth rates, and a minimum daily tree water deficit (TWD) close to 0) per site and year used to model the influence of climate conditions on daily growth

	S1200	S1700	S2000
2012	155–175	155–190	175–200
2013	120–140	175–190	170–200
2014	120–190	165–190	165–190

Long‐term climate–growth relations were tested with response functions using the packages dplR (Bunn *et al*., 2016) and treeclim (Zang & Biondi, 2015) in R, a form of principal components regression, which accounts for the collinearity in monthly climate predictors (Fritts *et al*., 1971; Briffa & Cook, 1990). Static and moving response functions were used to correlate site master chronologies of residuals resulting from ARMA modelling of the ring series (Holmes, 1994) to monthly mean temperature and total precipitation at a statistical significance of *P* < 0.05, using a bootstrap procedure with *N* = 1000 (Zang & Biondi, 2015).

All statistical analyses were performed using R (R Core Team, 2016); data manipulation was facilitated using the packages plyr (Wickham, 2011) and zoo (Zeileis & Grothendieck, 2005). Figures were generated using ggplot2 (Wickham, 2009) and egg (Baptiste, 2017).

## Results

### Microclimate

**Figure 1:**
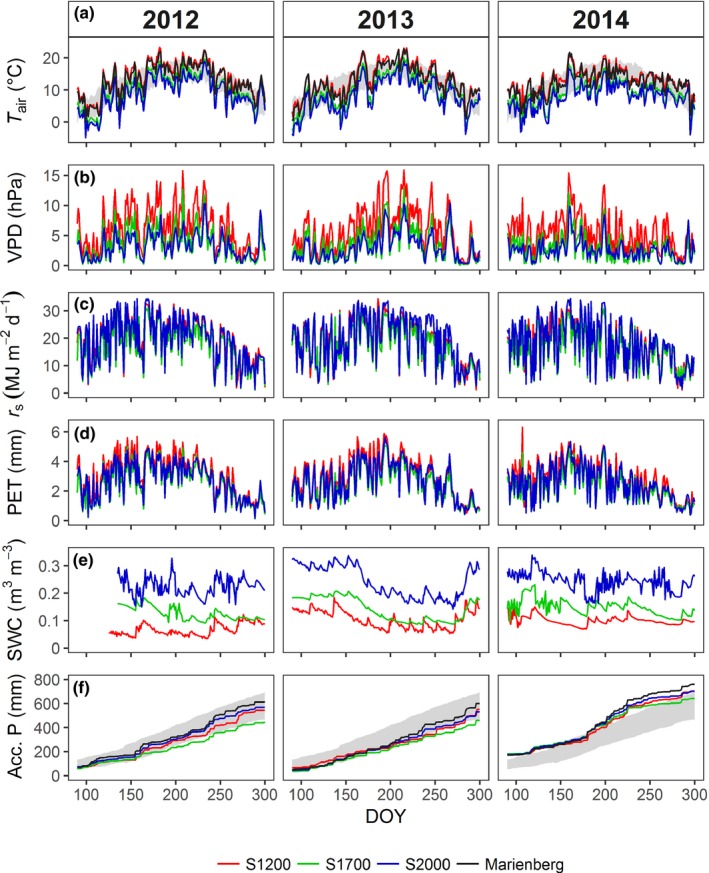
(a) Daily mean air temperature (*T*_air_), (b) vapor pressure deficit (VPD), (c) daily sums of global radiation (*r*_s_), (d), potential evapotranspiration (PET), (e) daily mean soil water content (SWC), and (f) accumulated daily precipitation sums (*P*_acc_) at the study sites S1200, S1700 and S2000 for the years 2012–2014. (a, e) These panels include 30 yr averages (1980–2010, ± SD indicated by the gray area) from the nearby climate station Marienberg (1310 m). DOY, day of year.

Yearly mean temperatures from 2012 to 2014 were more than 1°C higher than the 30 yr (1981–2010) average at the Marienberg station (Fig. 1a). Below‐average temperatures were only observed in winter 2013 and in July and August 2014. Precipitation corresponded to the long‐term average in 2012 and 2013. By contrast, precipitation in 2014 was > 100 mm higher than the long‐term average, with *c*. 2.5 times more snowfall from December to March than in the previous 2 yr (see Supporting Information Table S1a,b) and a rainy July and August (Fig. 1f). Temperature (Fig. 1a) and VPD (Fig. 1b) decreased more (yearly average of 3.7°C in total and 0.67°C per 100 m elevation difference) between S1200 and S1700 than between S1700 and S2000 (1.1 K in total and 0.39 K per 100 m). Precipitation increased with elevation by 8–19 mm per 100 m between S1200 and S1700 and by 22–30 mm per 100 m between S1700 and S2000 (the lowest elevational trend occurred in 2014). Mean global radiation was lowest at S1700 (Fig. 1c) as a result of increased shading by surrounding mountains. As a consequence, PET was highest at S1200 and lowest at S1700 (Fig. 1d). SWC also increased with elevation, with a stronger increase from S1700 to S2000 (Fig. 1e). At S2000 and S1700, snowmelt in March to April led to high SWC at the start of the growing season (Table S1a). Even though snow cover was inconsistent at S1200, especially in 2012/2013, low evapotranspiration in winter and precipitation in early spring still led to relatively high SWC at the start of growth. Growing season means of temperature, VPD, solar radiation, and PET were lower in 2014 than in the two previous years across all sites, while mean SWC and total precipitation were higher, mainly as a result of a cool and rainy July and August.

### Sap flow, intra‐annual radial growth, and TWD

**Figure 2:**
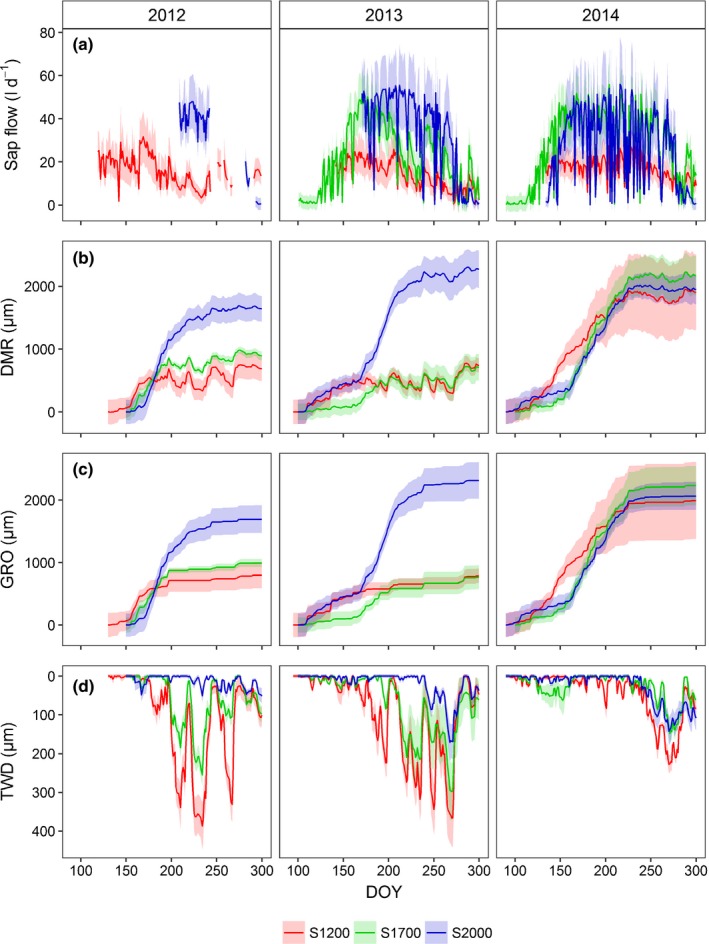
(a) Site average (± SE) of the daily sum of sap flow per tree, (b) radial stem variation (daily mean DMR), (c) potential growth (daily maximum GRO), and (d) daily minimum tree water deficit (TWD) at the study sites S1200, S1700 and S2000 for the growing seasons 2012–2014. DOY, day of year.

Daily sums of sap flow (averaged per site) generally increased early during the growing season, reached a maximum in late May to early June and decreased rapidly again in early autumn. Compared with S1200 and S1700, sap flow started to increase *c*. 20 d and reached its maximum *c*. 10 d later at S2000 in 2014 (Fig. 2a; sap flow data for spring 2012 and 2013 are partly missing as a result of sensor/logger malfunction). Sap flow decreased rapidly at all sites around DOY 270–280, corresponding to a substantial decrease of air temperature in 2013 and to a 2 wk dry period in 2014. Single days with low values, related to rainy weather conditions, were observed at all sites and more frequently in 2014 than in 2013. Maximum daily sums of sap flow were about the same at S1700 and S2000, but only half at S1200. While sap flow under fair‐weather conditions stayed rather constant between DOY 170 and DOY 260 at all sites in 2014, it decreased strongly and lastingly after DOY 200 at S1200 and S1700 in 2013 and after DOY 170 at S1200 in 2012. At S2000, no lasting decrease in sap flow was observed.

Dendrometer records and the estimated GRO curves showed a stronger yearly growth at S2000 than at the two lower sites in 2012 and 2013 (Fig. 2b, c). Growth at S1200 and S1700 ceased well before DOY 200, and stem radius shrank for several days during dry periods in these two summers. Correspondingly, TWD increased to > 200 μm, starting as early as DOY 175 at S1200 and S1700 in 2012 and 2013 (Fig. 2d). Such high TWD was reached at S2000 only after DOY 250 in autumn 2013. By contrast, yearly growth in 2014 exceeded the level of S2000 at S1700 and reached it at S1200. Hardly any decrease of DMR was observed at any site in 2014 until after DOY 240 and, correspondingly, TWD increased to values higher than 100 μm only in late summer/autumn.

**Figure 3:**
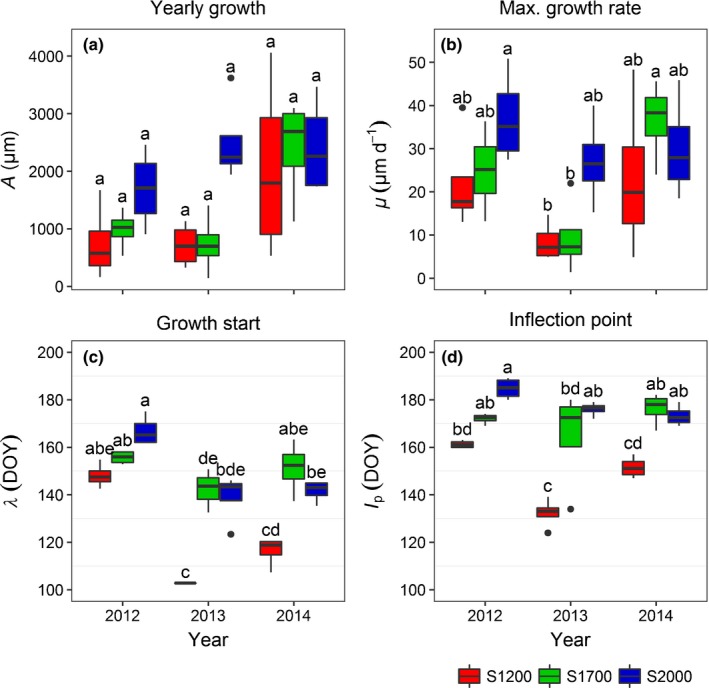
The parameters of the Gompertz functions fitted to the potential growth (GRO) curve of each tree grouped by measuring sites and years. (a) *A* is the upper asymptote corresponding to total yearly growth, (b) *μ* the maximum growth rate, (c) *λ* the start of growth, and (d) *I*_p_ the inflection point of the growth curves. The boxplots show median, interquartile range, and outliers. The letters ‘a’–‘e’ refer to significant differences between sites and years, tested with Tukey's honestly significant difference test. DOY, day of year.

Fitting Gompertz equations to the GRO curves confirmed higher annual increments at S2000 than at S1200 and S1700 in 2012 and 2013, while they were almost equal in 2014 (Fig. 3a). Maximum growth rates were constantly high at S2000 and lowest at S1200, although mean values in 2012 and 2014 were twice as high as in 2013 and extremely inconsistent at S1700, with values as low as at S1200 in 2013 and > S2000 in 2000 (Fig. 3b). The start of growth and the inflection point of the growth curves were always clearly earliest at S1200 and similar at S1700 and S2000 (Fig. 3c,d).

### Correlation of sap flow, daily radius changes, TWD vs microclimate

The best statistical model relating sap flow to microclimate showed site‐specific relationships of sap flow with PET and SWC (Table 3; Figs S1, S2). Sap flow increased strongly at low values of PET and SWC and levelled off at higher values; differences between sites were small for PET and larger for SWC. No significant correlation occurred between sap flow and SWC at S2000. During the growth period, the best models displayed nonsite‐specific relationships of DRC and TWD with PET and precipitation with a 1 d time lag (Table 3). DRC decreased initially with PET but increased again at higher values and increased nonlinearly with precipitation (Fig. S1c,d). TWD increased linearly with PET and decreased with precipitation at low and high values, while increasing in between (Fig. S1e,f). For the late summer period with no growth and DRC and TWD only depending on tree water status, the relationship of DRC with PET showed a far smaller increase of DRC at high values of PET than in the growth period (Fig. S2a). The DRC–precipitation relationship was site‐specific with the slope decreasing with elevation (Fig. S2b). TWD during late summer increased linearly and nonsite‐specifically with PET (Fig. S2c), decreased with precipitation (Fig. S2d) and decreased nonlinearly and site‐specifically with SWC (Fig. S2e). Using VPD and solar radiation instead of PET to characterize water demand resulted in models with mostly slightly higher variance explained (higher adjusted *r* ^2^; Table S2; Figs S3, S4). Both VPD and radiation had significant influence on sap flow, DRC and TWD. Variance explained by the GAMM models was high for the sap flow and late summer models and substantially lower for the growth period models; autocorrelation was strongest for the TWD models.

**Table 3:** Results of generalized additive mixed models to relate sap flow, daily radius change (DRC), and tree water deficit (TWD) to potential evaporation (PET), precipitation (*P*), and soil water content (SWC)

DOY	Sap flow			DRC						TWD					
	150–250 (2013 + 2014 only)			Growth period (see Table 2)			225–275			Growth period (see Table 2)			225–275		
*N*	561			278			458			278			459		
Adjusted *R* ^2^	0.87			0.37			0.60			0.17			0.61		
Phi	0.54			0.61			0.53			0.82			0.97		
	Linear estimate	Smoother‐edf	Sig. *P*‐value	Linear estimate	Smoother‐edf	Linear estimate	Linear estimate	Smoother‐edf	Sig. *P*‐value	Linear estimate	Smoother‐edf	Sig. *P*‐value	Linear estimate	Smoother‐edf	Sig. *P*‐value
Intercept	38.65		< 0.001	22.82		< 0.001	1.86		0.09	1.82		n.s.	74.41		< 0.001
PET					4.18	< 0.001		3.59	< 0.001	4.51		< 0.001	7.69		< 0.001
s(PET): S1200		5.31	< 0.001												
s(PET): S1700		5.34	< 0.001												
s(PET): S2000		4.79	< 0.001												
P_lag1					3.82	< 0.001					3.40	< 0.001			
P last 3													−0.68		< 0.001
s(P_lag1): S1200								1.74	< 0.001						
s(P_lag1): S1700								2.94	< 0.001						
s(P_lag1): S2000								1.58	< 0.001						
s(SWC_lag1): S1200		4.90	< 0.001											3.46	< 0.001
s(SWC_lag1): S1700		4.97	< 0.001											4.85	< 0.001
s(SWC_lag1): S2000		< 0.01	n.s.											0.0001	n.s.

DOY, time period included in the model; *N*, number of observations (measuring days); adj. *r* ^2^, variance explained; phi, a measure of autocorrelation. For linear relations the estimate of intercept and slope (‘linear‐estimate’), for nonlinear smoothers the estimated degrees of freedom (describing the shape of the smoother, ‘smoother‐edf’). are displayed. ‘_lag1’ following the climate variables indicates when using a 1 d lag period provided better results, ‘P last 3’ is the sum of precipitation of the current and the two previous days. In case site‐specific relationships were found, the edf‐value is given for each site.

### Long‐term climate–growth relationship

Mean ring width was highest at the S1200 and S2000 sites and lowest at S2200 and S1100, with generally high standard deviations (see Table 4). Mean sensitivity, an indicator of relative change between consecutive rings (Fritts, 1976), decreased with elevation from 0.51 at S1100 to 0.17 at S2000, but increased again to 0.34 at the forest line (S2200). First‐order autocorrelation, describing the correlation of tree ring width with the previous year, was highest at S1200 and S1700. Rather high signal‐to‐noise ratios and expressed population signal values clearly above the commonly accepted threshold of 0.85 at all sites indicate strong, coherent stand‐level climate signals (Cook *et al*., 1990; Speer, 2010).

**Table 4:** Chronology statistics of selected stands along an elevation gradient

Site	Trees^a^ (*n*)	Age^b^ (yr)	Mean ring width (μm)	MS^c^	Autocorr^c^	SNR^c^	EPS^c^	OIC^c^
S1100	10/7	120.6 ± 3.6	1080 ± 930	0.51	0.14	21.87	0.96	0.84
S1200	15/15	45.5 ± 3.0	2630 ± 1860	0.39	0.27	30.33	0.97	0.79
S1700	15/15	109.1 ± 4.3	1480 ± 720	0.26	0.44	24.81	0.96	0.73
S2000	15/13	74.2 ± 17.3	2190 ± 870	0.17	0.34	14.44	0.94	0.63
S2200	10/7	92.4 ± 56.3	1350 ± 480	0.34	0.22	9.08	0.90	0.57

Trees, number of trees sampled/included in the site chronology; MS, mean sensitivity; Autocorr, first‐order autocorrelation; SNR, signal‐to‐noise ratio; EPS, expressed population signal; OIC, overall interseries correlation. Age and mean ring width are site averages ± SD (for trees included in the site chronology).

a Each tree was cored twice at opposite sides and parallel to the contour line.

b Cambial age at *c*. 1.3 m above ground.

c Calculated before prewhitening, that is, removing of serial autocorrelation.

**Figure 4:**
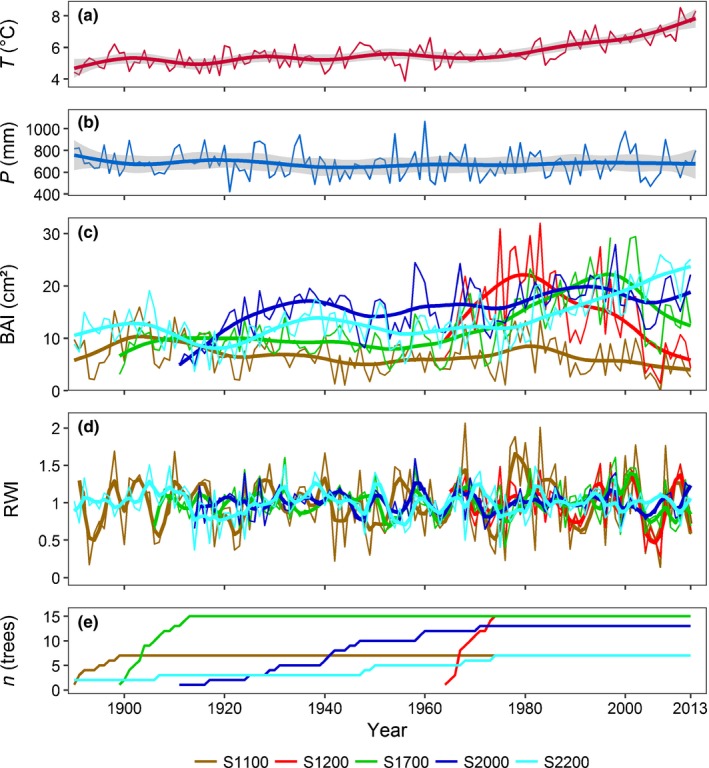
(a) Time course of mean annual air temperature (*T*), (b) total precipitation (*P*) at the Marienberg climate station (1310 m) and (c) site averages of basal area increment (BAI), (d) ring width index (RWI; describing the residual chronologies after removing long‐term trends and used for calculating climate–growth relations) and (e) sampling depth *n*, at the five measuring sites from 1890 to 2013 (as trees were cored in summer 2014). Thin lines show yearly data; thick lines are 25* *yr (for *T*,* P* and BAI) and 5 yr (for RWI) smoothers; gray areas in (a) and (b) display 95% confidence regions.

The substantial increase of air temperatures, especially since 1980, caused different growth responses along the elevation transect (Fig. 4a–c). Basal area increment (BAI) was consistently low at S1100 since the 1920s, with comparatively high values in the more humid late 1970s. At S1200, growth rates decreased rapidly after extremely high initial values and were the second lowest of all sites since the 1990s. At S1700, growth increased strongly from the 1960s until the 1990s, but decreased rapidly afterwards. Growth rate at S2000 was consistently high since the 1930s. After some fluctuations around a constant average from the 1900s to the 1960s, growth increased strongly at S2200 since the 1970s and was highest in the last 20 yr. Fig. 4(d) depicts the residual chronologies after removing long‐term trends used for calculating climate–growth relations. Contrasting growth trends of S2000 and S2200 compared with the lower‐elevation sites observed, especially in recent decades, indicate an opposing influence of climatic factors.

**Figure 5:**
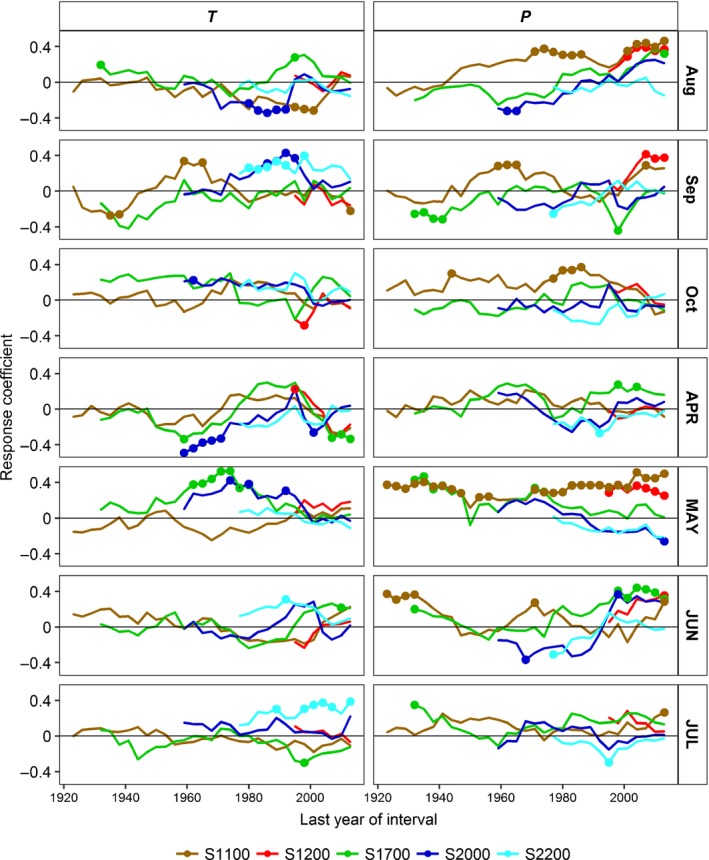
Moving 25 yr response function analysis between residual chronologies and monthly mean temperature (*T*) and total precipitation (*P*) for periods with a sampling depth of at least five trees per site for selected months in the previous (lower case) and current (capital letters) year. Full circles indicate significant relationships at *P* < 0.1.

Response functions revealed that tree ring width was directly correlated with spring and early summer precipitation at the three lower elevation sites (Figs 5, S5). At S1100 and S1200, precipitation in January (snowfall) and in the previous late August and September also had a positive effect on radial growth. S1700 showed a positive T‐response for May and the previous October and a negative one for the previous December. At the two higher‐elevation sites, April temperature and, for S2000, also previous July ‘temperature’ were indirectly correlated with tree ring width, while current July and previous September temperatures had a positive influence at S2200. Precipitation had no significant influence on tree ring width at S2000 and S2200. A moving‐response function revealed that the positive influence of the previous August and September precipitation, current May precipitation (S1100 and S1200) and June precipitation (S1200, S1700, and S2000), and the negative response on February and March precipitation (S2000 and S2200) were increasing in recent decades. By contrast, the positive growth response on January precipitation (S1100 and S1200), previous September temperature (at S2000 and S2200) and current May temperature (at S1700 and S2000) decreased recently (Fig. 5, S6). Extremely warm and dry summers, such as in 1976 or 2003, influenced growth negatively at S1100, S1200 and S1700, while trees at the two higher elevations hardly reacted or even showed an increase of growth (S2200 in 1976). By contrast, colder and humid summers, such as in 1975, led to positive growth reactions at the three lower sites and a negative one at the forest line (Fig. 4; Table S3).

### Estimation of current and future growth conditions of larch in the Alpine region

**Figure 6:**
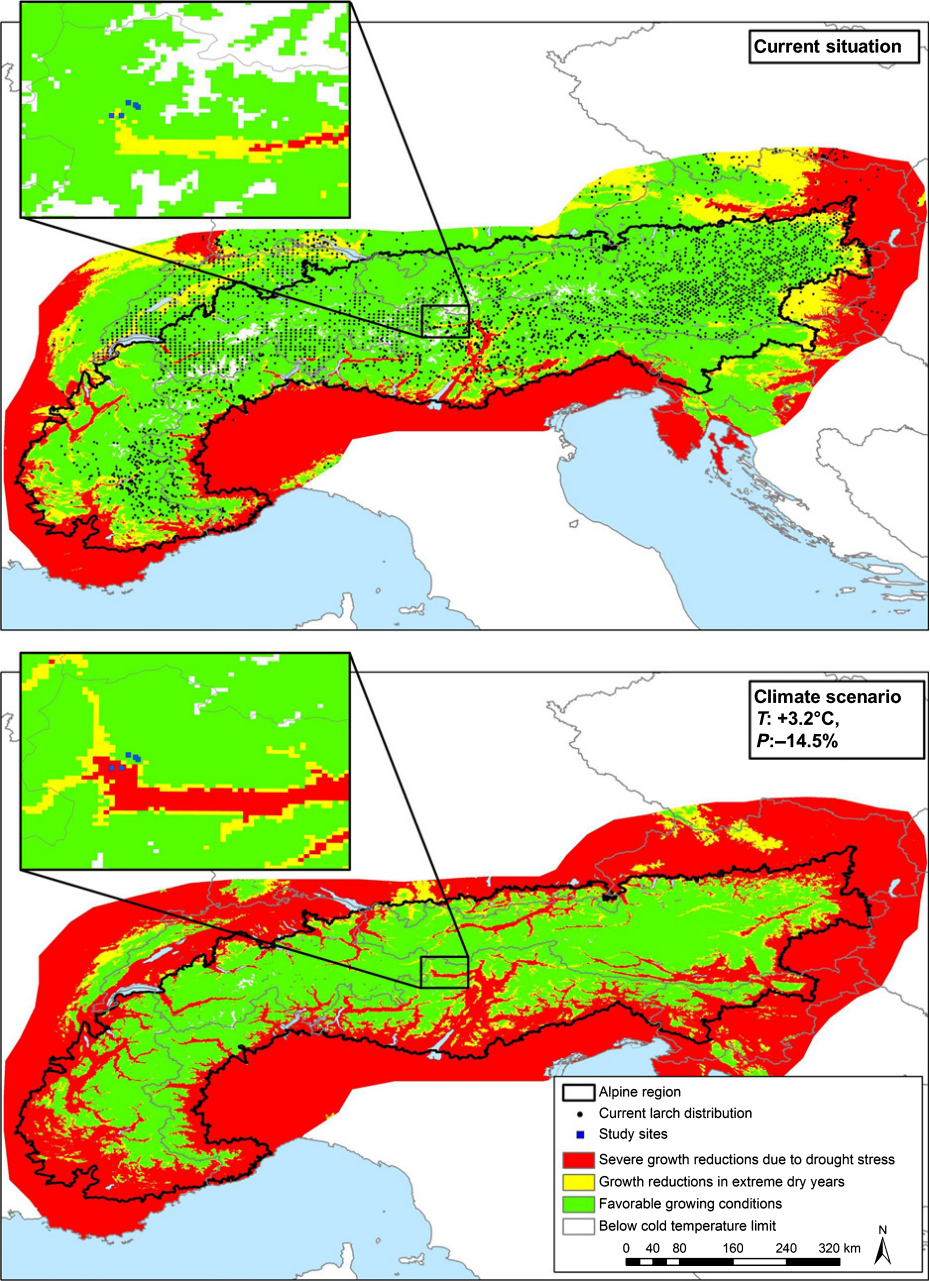
Current and future (2069–2098, based on Gobiet *et al*., 2014) climatic growth conditions of European larch in the Alps and surrounding regions based on an upscaling of our measurements. Growth conditions were estimated based on climatic water deficit (precipitation minus potential evaporation) and a minimum yearly average temperature of −2.5°C for larch survival (Kölling, 2007). Current conditions at sites S1100 and S1200 correspond to the red area, S1700 to the yellow area, and S2000 and S2200 to the green area in the maps. Nonclimatic factors of larch distribution, such as land use, soil conditions, competition, and dissemination were not accounted for in the upscaling and the climate‐change scenario. Current larch distribution is from the European Forest Institute ( https://www.efi.int/knowledge/maps/treespecies) and Info Flora ( www.infoflora.ch) for Switzerland.

Upscaling the results of our transect sites to the Alpine range and its surroundings (Fig. 6) indicated good growing conditions for most of the Alps (except for some southern valley floors and lower areas in the southwest) and its northern foothills. The scenario with increased temperature and reduced precipitation displayed a major increase of areas with severe, drought‐induced growth restrictions in most major valleys and lowlands in the Alpine range and its foothills. By contrast, at higher elevations in the central Alps, areas with favorable growth conditions will increase.

## Discussion

By measuring sap flow, stem radius variation, and tree ring widths of larch along an elevation gradient, we covered climate conditions ranging from the dry edge of this species’ distribution at low elevation to the rather cool and humid environment at the forest line (Körner, 2007a,b; King *et al*., 2013). We expected growth limitations by water shortage at low elevation and by low temperatures at the forest line and near‐optimal conditions in between. By contrast, we observed intra‐annual and long‐term growth limitations up to an elevation of 1700 m, while growth rates at 2000 m hardly responded to variations in temperature or precipitation. Remarkably, highest growth rates during the last 10 yr occurred at the forest line at 2300 m, which indicates an ongoing shift of favorable growth conditions to higher elevations in inner‐alpine environments. With a geographic information system‐based upscaling, we demonstrated that the elevation gradient mirrored future conditions of larger regions in and around the Alps. While areas with severe growth restrictions as a result of limited water availability (corresponding to our low‐elevation sites) are currently restricted to some valleys within the Alps and their foothills, they will increase strongly by the end of the century. Increasing temperatures and decreasing precipitation (EEA, 2012; Elkin *et al*., 2013; APCC, 2014) will then cause increased drought stress.

### Sap flow

European larch is considered an anisohydric tree species, which does not show restrictive stomata regulation (Anfodillo *et al*., 1998; Leo *et al*., 2014). It compensates for a short growing season (as a result of its deciduous needle leaves) by maintaining its photosynthetic activity also during drier conditions (Lévesque *et al*., 2013). Consequently, Anfodillo *et al*. (1998) observed a strong coupling of sap flow with VPD (i.e. evaporative demand) in larch and no reduction in sap flow during short summer droughts (i.e. a minor reduction of water supply). Accordingly, at S2000, sap flow was strongly influenced by day‐to‐day weather conditions, but did not show any lasting reduction during summer. By contrast, we observed a reduction of sap flow when dry periods reduced (initially high) SWC substantially in the summer of 2012 and 2013 at S1200, but also at S1715 (i.e. upper montane/low subalpine belt; Fig. 2a). Leo *et al*. (2014) found a similar reduction of sap flow in larch in a rain exclusion experiment, where daily sap flow decreased by 25% in sheltered compared with control trees. The shelter prevented a refilling of soil water reservoirs after a long drought in April diminished the effects of snowmelt. In our study, drought‐induced reductions in sap flow were only absent in 2014, which was a humid year with > 100 mm more annual precipitation than the 30 yr average.

The observed saturation of the sap flow–PET and sap flow–VPD relationships (Figs S1, S3) corresponds to findings by Leo *et al*. (2014), Anfodillo *et al*. (1998), and Badalotti *et al*. (2000). While the relationship between sap flow and radiation is described as linear in the literature (Matyssek *et al*., 2009), we found saturation at a solar radiation of 15 (at S1200) to 25 MJ m^−2^ d^−1^(at S2000), which is an effect of open stand structure (Larcher, 2003). Leo *et al*. (2014) found a stronger reaction of sap flow to radiation in unsheltered (and therefore less water‐limited) conditions as we found the strongest response at our most humid site, S2000. The lack of a significant sap flow*–*SWC correlation at S2000 indicates sufficient water supply at high elevation, while sap flow at the two lower sites was limited by soil water availability. The different shape of the sap flow–SWC relationship at the two lower sites is a result of generally higher SWC and sap flow at S1700 compared with S1200.

### Stem radius variations, TWDs, and intra‐annual growth

Lower annual increments measured at S1200 and S1700 in 2012 and 2013 were mainly caused by an earlier termination of the growth period around DOY 190, while radial growth continued until *c*. DOY 225 at S2000 in 2012 and 2013 and at all sites in the more humid year 2014 (Fig. 2). The cessation of growth at S1200 and S1700 coincided with a reduction in SWC and sap flow and was followed by periods of further reduction in stem radius (DMR) and high TWD, which were interrupted by occasional rainfall. Together, this provides strong evidence for the dependence of radial growth on water availability at S1200 and S1700 (Bréda *et al*., 2006). Higher TWD values after DOY 240, which were also measured at S2000 in 2013 and 2014, did not affect either growth of the current year (the growing season was already finished) or growth of the following year. In larch, an early cessation of radial growth as a result of limited water availability has also been observed by Oberhuber *et al*. (2014) and, similarly, in *P. abies* and *Pinus sylvestris* (Pichler & Oberhuber, 2007; Levanič *et al*., 2009; Thabeet *et al*., 2009), while Moser *et al*. (2010) found hardly any effect of elevation on the end of the larch growing season under nonwater‐limited conditions. Maximum growth rates at S2000 appeared to be less influenced by variations in temperature and precipitation than at S1200 and S1700 (Fig. 3b). The initial low growth rates in 2013, especially at S1700, were probably caused by low temperatures in May, while decreasing growth rates at S1200 and S1700 in June 2012 and 2013 were presumably a consequence of water limitation. Temperature appeared to be the main reason for growth patterns observed in the early growing season when water supply was adequate as a result of snowmelt and spring precipitation (*λ*; Fig. 3c): warmer spring temperatures at S1200 led to a considerably earlier increase in growth rate, while spring temperatures and the start of radial growth at S1700 and S2000 were similar. Moser *et al*. (2010) found a delay of 3–4 d per 100 m elevation in the onset of radial growth in larch.

We observed no difference between sites in the correlations of daily stem radius changes (DRC) and TWD vs PET (or VPD and radiation) and precipitation during the growth period (Table 3; Figs S1c–f, S3d–i). The lack of elevational differences indicates a sufficient water supply during the main growing season at all study sites. By contrast, water limitations of various extent during late summer induced site‐specific correlations of DRC–precipitation and TWD*–*SWC (Table 3; Figs S2, S4). The correlation of DRC and TWD to precipitation (mostly of previous days) shows the sensitive response of stem radius to rainfall events. The lack of a correlation of DRC*–*SWC, even during dry late summer, indicates a buffering of hydraulics by stem water reservoirs (Schulze *et al*., 1985; Oberhuber *et al*., 2015b) and/or uptake of water from deeper soil layers and cracks (Valentini *et al*., 1994; Brooks *et al*., 2010). Soil moisture in the uppermost soil layers thus may not sufficiently reflect the water supply of trees (Oren & Pataki, 2001; Zweifel *et al*., 2006). We found a decrease of DRC with PET (for most of the range of PET) and a linear decrease of DRC with VPD at all sites, similar to Oberhuber *et al*. (2014). The strong correlation of DRC as well as sap flow with PET and its principal driving force VPD indicates a close coupling of transpiration, sap flow, and diurnal stem shrinkage (Steppe *et al*., 2006). Decreasing TWD at high PET/VPD might be a critical adjustment considering increasing temperatures as a result of climate change.

### Long‐term effects of climatic conditions on tree growth

Long‐term climate–growth relations from tree ring width series corresponded well to short‐term sap flow and dendrometer records. They displayed a dependence of growth on precipitation up to an elevation of > 1700 m and a decrease of yearly growth at low elevation with increasing temperatures in recent decades. Positive growth responses to precipitation, negative responses to temperature, and/or limited growth rates at lower elevation (1400 m) were also reported for larch, *P. sylvestris*,* P. abies*, and *Abies alba* in other dry alpine areas (Affolter *et al*., 2010; Eilmann & Rigling, 2012; King *et al*., 2013; Lévesque *et al*., 2013, 2016a; Schuster & Oberhuber, 2013a; Swidrak *et al*., 2013). While we observed positive temperature responses of larch growth only at our highest elevations (S2000 and S2200), they were also found below, providing sufficient water supply (e.g., see Hartl‐Meier *et al*., 2014). In a multispecies overview for Europe, Babst *et al*. (2013) found negative temperature and positive precipitation correlations to be typical for lower elevations in inner‐Alpine dry valleys. They observed temperature responses above and precipitation responses below a threshold, decreasing from the forest line at 2250 m at 42°N to 1000 m at 49°N and further to sea level at 60°N. Accordingly, a precipitation response should be expected at elevations below 1500 m in our research area (latitude 46°N). However, we actually observed a precipitation response up to 1700 m and a temperature response only above 2000 m, which is an effect of the particularly dry climatic conditions at the study site.

Additional information provided by long‐term tree ring records compared with high‐resolution, but short‐term dendrometer measurements, are the carry‐over effects of late summer and autumn conditions on tree ring width of the following year (Lévesque *et al*., 2013, 2016b; Schuster & Oberhuber, 2013b), the multi‐year effects of extreme events, and long‐term trends in growth and climate–growth relations. A growth reduction lasting several years (as observed after the dry summers of 2003 and 2004 at S1100, S1200, and S1700) was also found for larch by Eilmann & Rigling (2012) after the end of irrigation at a reforestation site in the Valais. Positive and increasing growth responses of high‐elevation forests to rising temperatures, as observed at S2000 and S2200, are regularly found not only in larch, but also in *P. abies*,* P. cembra*, and *Pinus uncinata* (Carrer *et al*., 1998; Rolland *et al*., 1998; Frank & Esper, 2005; Carrer & Urbinati, 2006). As atmospheric CO_2_ and N‐fertilization effects and forest management effects do not explain the decadal fluctuations and regional patterns, Rolland *et al*. (1998) attributed the increase of radial growth in high‐elevation forests to climate change. At low elevation, the increasing precipitation sensitivity of radial growth in recent decades might be caused by rising temperatures, although an increase of water competition in denser stands with larger trees should also be considered (Schuster & Oberhuber, 2013a). Regular outbreaks of Larch budmoth (*Zeiraphera diniana*) were found to reduce tree ring width and disturb climate–growth relations of larch (Weber, 1997; Esper *et al*., 2007; Baltensweiler *et al*., 2008; Büntgen *et al*., 2009; Battipaglia *et al*., 2014; Saulnier *et al*., 2017). Following the methods suggested by Büntgen *et al*. (2009) and Saulnier *et al*. (2017), we found evidence of larch budmoth outbreaks at S1700, S2000, and S2200 (in the years 1923, 1947, 1957, 1968, 1975 and 1984) but not at the lower‐elevation sites; excluding those years did not improve climate–growth correlations.

### Conclusion

Our data confirm that climate change leads to increasing growth rates of trees at elevations close to the forest line. In the densely populated and managed Alps, a possible expansion of larch to higher elevations will, however, depend not only on climatic conditions, but also on the further management or abandonment of alpine pastures and meadows (Tasser *et al*., 2017). Also, more competitive species such as *P. abies* might move upwards and invade larch–*P. cembra* forests (Wolfslehner *et al*., 2011). While growth limitations of larch upon drought were reported in the literature mostly based on long‐term tree ring data, we could demonstrate short‐term reductions of sap flow and found stem growth limitations up to the lower subalpine belt and thus at higher‐than‐expected elevations. With increasing temperatures and decreasing precipitation, these limitations will probably apply not only to inner alpine dry valleys, but also to a larger geographical range in the future. For forestry purposes, decreasing growth rates might limit the future forest‐economical suitability of larch for large areas of the Alps. However, we also found that humid years, such as 2014, led to high growth rates even at low elevations, indicating that larch will still play an important role as a stabilizing tree species in mixed forests over the coming decades.

## Author contributions

N.O., C.N., E.T., W.O., S.M. and U.T. planned and designed the research. N.O. and A.M. conducted field measurements and data analysis, E.T. and N.O. performed the upscaling. N.O. wrote the manuscript. C.N., E.T., W.O., S.M. and U.T. edited the manuscript.

## Supporting information

Please note: Wiley Blackwell are not responsible for the content or functionality of any Supporting Information supplied by the authors. Any queries (other than missing material) should be directed to the *New Phytologist* Central Office.

**Fig. S1** Regression lines and smoothers for the general additive mixed models between sap flow, daily radius change as well as tree water deficits and potential evaporation as well as water availability expressed by precipitation and/or soil water content.
**Fig. S2** Regression lines and smoothers for the late summer period (DOY 225–275) for the general additive mixed models between daily radius change as well as tree water deficits and potential evaporation as well as water availability expressed by precipitation and/or soil water content.
**Fig. S3** Regression lines and smoothers for the general additive mixed models between sap flow, daily radius change as well as tree water deficits and vapor pressure deficit, global radiation, precipitation, and soil water content.
**Fig. S4** Regression lines and smoothers for the general additive mixed models between daily radius change as well as tree water deficits and vapor pressure deficit, global radiation, precipitation, and soil water content for the late summer period (DOY 225–275).
**Fig. S5** Static response function analysis between residual chronologies and monthly mean temperature and total precipitation.
**Fig. S6** Moving 25 yr response function analysis between residual chronologies and monthly mean temperature and total precipitation.
**Table S1** Precipitation and snow cover for the three winter seasons preceding the sap flow/dendrometer measuring seasons as well as long‐term (1980–2010) averages; winter precipitation (November to March) for 2011/2012, 2012/2013, and 2013/2014 as well as long‐term (1980–2010) averages and standard deviation at the climate station Marienberg/Monte Maria
**Table S2** Results of generalized additive mixed models to relate sap flow, daily radius change, and tree water deficits to vapor pressure deficit, global radiation, precipitation, and soil water content
**Table S3** Growth reactions of trees to selected pointer years

## References

[nph15348-bib-0001] Affolter P , Büntgen U , Esper J , Rigling A , Weber P , Luterbacher J , Frank D . 2010 Inner Alpine conifer response to 20th century drought swings. European Journal of Forest Research 129: 289–298.

[nph15348-bib-0002] Allen RG , Pereira LS , Raes D , Smith M . 1998 Crop evapotranspiration – guidelines for computing crop water requirements – FAO Irrigation and drainage paper 56. Rome, Italy: FAO – Food and Agriculture Organization of the United Nations.

[nph15348-bib-0003] Anfodillo T , Rento S , Carraro V , Furlanetto L , Urbinati C , Carrer M , Anfodillo T . 1998 Tree water relations and climatic variations at the alpine timberline: seasonal changes of sap flux and xylem water potential in *Larix decidua* Miller, *Picea abies* (L.) Karst. and *Pinus cembra* L. Annals of Forest Science 55: 159–172.

[nph15348-bib-0004] APCC . 2014 Österreichischer Sachstandsbericht Klimawandel 2014 (AAR14). Austrian Panel on Climate Change (APCC) , ed. Vienna, Austria: Verlag der Österreichischen Akademie der Wissenschaften.

[nph15348-bib-0005] Auer I , Böhm R , Jurkovic A , Lipa W , Orlik A , Potzmann R , Schöner W , Ungersböck M , Matulla C , Briffa K *et al* 2007 HISTALP—historical instrumental climatological surface time series of the Greater Alpine Region. International Journal of Climatology 27: 17–46.

[nph15348-bib-0006] Babst F , Poulter B , Trouet V , Tan K , Neuwirth B , Wilson R , Carrer M , Grabner M , Tegel W , Levanic T *et al* 2013 Site‐ and species‐specific responses of forest growth to climate across the European continent. Global Ecology and Biogeography 22: 706–717.

[nph15348-bib-0007] Badalotti A , Anfodillo T , Grace J . 2000 Evidence of osmoregulation in *Larix decidua* at Alpine treeline and comparative responses to water availability of two co‐occurring evergreen species. Annals of Forest Science 57: 623–633.

[nph15348-bib-0008] Baltensweiler W , Weber UM , Cherubini P . 2008 Tracing the influence of larch‐bud‐moth insect outbreaks and weather conditions on larch tree‐ring growth in Engadine (Switzerland). Oikos 117: 161–172.

[nph15348-bib-0009] Baptiste A . 2017 egg: (fragile) extensions for ggplot2. [WWW document] URL https://CRAN.R-project.org/package=egg [accessed 10 October 2016].

[nph15348-bib-0010] Battipaglia G , Büntgen U , McCloskey SPJ , Blarquez O , Denis N , Paradis L , Brossier B , Fournier T , Carcaillet C . 2014 Long‐term effects of climate and land‐use change on larch budmoth outbreaks in the French Alps. Climate Research 62: 1–14.

[nph15348-bib-0011] Becker A , Körner C , Brun J‐J , Guisan A , Tappeiner U . 2007 Ecological and land use studies along elevational gradients. Mountain Research and Development 27: 58–65.

[nph15348-bib-0012] Begueria S , Serrano V , Sawasawa H . 2017 SPEI: calculation of standardised precipitation‐evapotranspiration index. https://CRAN.R‐project.org/package=SPEI [accessed 15 September 2016].">[WWW document] URL https://CRAN.R‐project.org/package=SPEI [accessed 15 September 2016].

[nph15348-bib-0013] Beniston M . 2005 Mountain climates and climatic change: an overview of processes focusing on the European Alps. Pure and Applied Geophysics 162: 1587–1606.

[nph15348-bib-0014] Biondi F , Qeadan F . 2008 A theory‐driven approach to tree‐ring standardization: defining the biological trend from expected basal area increment. Tree‐Ring Research 64: 81–96.

[nph15348-bib-0015] Böhm R , Auer I , Brunetti M , Maugeri M , Nanni T , Schöner W . 2001 Regional temperature variability in the European Alps: 1760–1998 from homogenized instrumental time. International Journal of Climatology 1801: 1779–1801.

[nph15348-bib-0016] Bréda N , Huc R , Granier A , Dreyer E . 2006 Temperate forest trees and stands under severe drought: a review of ecophysiological responses, adaptation processes and long‐term consequences. Annals of Forest Science 63: 625–644.

[nph15348-bib-0017] Briffa K , Cook E . 1990 Methods of response function analysis In: Cook E, Kairiukstis L, eds. Methods of dendrochronology. Dordrecht, the Netherlands: Kluwer Academic Publishers, 165–178.

[nph15348-bib-0018] Brooks JR , Barnard HR , Coulombe R , McDonnell JJ . 2010 Ecohydrologic separation of water between trees and streams in a Mediterranean climate. Nature Geoscience 3: 100–104.

[nph15348-bib-0019] Bunn A , Korpela M , Biondi F , Campelo F , Mérian P , Qeadan F , Zang C . 2016 dplR: Dendrochronology Program Library in R. [WWW document] URL https://CRAN.R-project.org/package=dplR [accessed 30 September 2015].

[nph15348-bib-0020] Büntgen U , Frank D , Liebhold A , Johnson D , Carrer M , Urbinati C , Grabner M , Nicolussi K , Levanic T , Esper J . 2009 Three centuries of insect outbreaks across the European Alps. New Phytologist 182: 929–941. 19383093 10.1111/j.1469-8137.2009.02825.x

[nph15348-bib-0021] Carrer M , Anfodillo T , Urbinati C , Carraro V . 1998 High‐altitude forest sensitivity to global warming: results from long‐term and short‐term analyses in the eastern Italian alps In: Beniston M, Innes J, eds. The impacts of climate variability on forests. *Lecture notes in earth sciences*,* vol. 74* Berlin/Heidelberg, Germany: Springer, 171–189.

[nph15348-bib-0022] Carrer M , Urbinati C . 2006 Long‐term change in the sensitivity of tree‐ring growth to climate forcing in *Larix decidua* . New Phytologist 170: 861–871. 16684244 10.1111/j.1469-8137.2006.01703.x

[nph15348-bib-0023] Čermák J , Kučera J , Nadezhdina N . 2004 Sap flow measurements with some thermodynamic methods, flow integration within trees and scaling up from sample trees to entire forest stands. Trees‐Structure and Function 18: 529–546.

[nph15348-bib-0024] Ciccarelli N , Von Hardenberg J , Provenzale A , Ronchi C , Vargiu A , Pelosini R . 2008 Climate variability in north‐western Italy during the second half of the 20th century. Global and Planetary Change 63: 185–195.

[nph15348-bib-0025] Cook E , Briffa K , Shiyatov S , Mazepa V . 1990 Tree‐ring standardization and growth‐trend estimation In: Cook E, Kairiukstis L, eds. Methods of dendrochronology. Applications in the environmental sciences. Dordrecht, the Netherlands: Kluwer Academic Publishers, 104–123.

[nph15348-bib-0026] Cook E , Holmes R . 1984 Program ARSTAN user manual: laboratory of tree ring research. Tucson, AZ, USA: University of Arizona.

[nph15348-bib-0027] Della Chiesa S , Bertoldi G , Niedrist G , Obojes N , Endrizzi S , Albertson JD , Wohlfahrt G , Hörtnagl L , Tappeiner U . 2014 Modelling changes in grassland hydrological cycling along an elevational gradient in the Alps. Ecohydrology 7: 1453–1473.

[nph15348-bib-0028] Deslauriers A , Rossi S , Anfodillo T . 2007 Dendrometer and intra‐annual tree growth: what kind of information can be inferred? Dendrochronologia 25: 113–124.

[nph15348-bib-0029] EEA . 2012 Climate change, impacts and vulnerability in Europe 2012. An indicator‐based report. Copenhagen, Denmark: EEA, Copenhagen.

[nph15348-bib-2004] Ehrenberger W , Rüger S , Fitzke R , Vollenweider P , Günthardt‐Goerg M , Kuster T , Zimmermann U , Arend M . 2012 Concomitant dendrometer and leaf patch pressure probe measurements reveal the effect of microclimate and soil moisture on diurnal stem water and leaf turgor variations in young oak trees. Functional Plant Biology 39: 297. 10.1071/FP11206 32480782

[nph15348-bib-0030] Eilmann B , Rigling A . 2012 Tree‐growth analyses to estimate tree species’ drought tolerance. Tree Physiology 32: 178–187. 22363071 10.1093/treephys/tps004

[nph15348-bib-0031] Elkin C , Gutiérrez AG , Leuzinger S , Manusch C , Temperli C , Rasche L , Bugmann H . 2013 A 2 °C warmer world is not safe for ecosystem services in the European Alps. Global Change Biology 19: 1827–1840. 23505061 10.1111/gcb.12156

[nph15348-bib-0032] Esper J , Buntgen U , Frank DC , Nievergelt D , Liebhold A . 2007 1200 years of regular outbreaks in Alpine insects. Proceedings of the Royal Society B: Biological Sciences 274: 671–679. 10.1098/rspb.2006.0191 PMC2197206 17254991

[nph15348-bib-0033] Ewers BE , Oren R , Albaugh TJ , Dougherty PM . 1999 Carry‐over effects of water and nutrient supply on water use of *Pinus taeda* . Ecological Applications 9: 513–525.

[nph15348-bib-0034] Falk W , Bachmann‐Gigl U , Kölling C . 2012 Die Europäische Lärche im Klimawandel In: Schmidt O, ed. Beiträge zur Europäischen Lärche. Freising, Germany: Bayerische Landesanstalt für Wald und Forstwirtschaft, 19–27.

[nph15348-bib-0035] Fisher RA , Koven CD , Anderegg WRL , Christoffersen BO , Dietze MC , Farrior CE , Holm JA , Hurtt GC , Knox RG , Lawrence PJ *et al* 2018 Vegetation demographics in Earth System Models: a review of progress and priorities. Global Change Biology 24: 35–54. 28921829 10.1111/gcb.13910

[nph15348-bib-0036] Fontana V , Radtke A , Walde J , Tasser E , Wilhalm T , Zerbe S , Tappeiner U . 2014 What plant traits tell us: consequences of land‐use change of a traditional agro‐forest system on biodiversity and ecosystem service provision. Agriculture, Ecosystems and Environment 186: 44–53.

[nph15348-bib-0037] Frank D , Esper J . 2005 Temperature reconstructions and comparisons with instrumental data from a tree‐ring network for the European Alps. International Journal of Climatology 25: 1437–1454.

[nph15348-bib-0038] Fritts HC . 1976 Tree rings and climate. London, UK: Academic Press.

[nph15348-bib-0039] Fritts HC , Blasing TJ , Hayden BP , Kutzbach JE . 1971 Multivariate techniques for specifying tree‐growth and climate relationships and for reconstructing anomalies in paleoclimate.pdf. Journal of Applied Meteorology 10: 845–864.

[nph15348-bib-0040] Ganthaler A , Bauer H , Gruber A , Mayr M , Oberhuber W , Mayr S . 2014 Effects of the needle bladder rust (*Chrysomyxa rhododendri*) on Norway spruce: implications for subalpine forests. European Journal of Forest Research 133: 201–211.

[nph15348-bib-0041] Gobiet A , Kotlarski S , Beniston M , Heinrich G , Rajczak J , Stoffel M . 2014 21st century climate change in the European Alps – a review. Science of the Total Environment 493: 1138–1151. 23953405 10.1016/j.scitotenv.2013.07.050

[nph15348-bib-0042] Gower ST , Richards JH . 1990 Larches: deciduous conifers in an evergreen world. BioScience 40: 818–826.

[nph15348-bib-0043] Gruber A , Wieser G , Oberhuber W . 2009 Intra‐annual dynamics of stem CO_2_ efflux in relation to cambial activity and xylem development in *Pinus cembra* . Tree Physiology 29: 641–649. 19203979 10.1093/treephys/tpp001 PMC3013296

[nph15348-bib-0044] Hartl‐Meier C , Dittmar C , Zang C , Rothe A . 2014 Mountain forest growth response to climate change in the Northern Limestone Alps. Trees 28: 819–829.

[nph15348-bib-0045] Holmes R . 1994 Dendrochronology program library user's manual. Laboratory of Tree‐Ring Research. Tucson, AZ, USA: University of Arizona.

[nph15348-bib-0046] IPCC . 2014 Climate Change 2014: Impacts, adaptation, and vulnerability. Part B: regional aspects. Contribution of Working Group II to the Fifth Assessment Report of the Intergovernmental Panel on Climate Change (Barros VR, Field CB, Dokken DJ, Mastrandrea MD, Mach KJ, Bilir TE, Chatterjee M, Ebi KL, Estrada YO, Genova RC *et al*, Eds.). Cambridge, UK and New York, NY, USA: Cambridge University Press.

[nph15348-bib-0047] Kahm M , Hasenbrink G , Lichtenberg‐Fraté H , Ludwig J , Kschischo M . 2010 Grofit: fitting biological growth curves with R. Journal of Statistical Software 33: 1–21.20808728

[nph15348-bib-0048] King GM , Gugerli F , Fonti P , Frank DC . 2013 Tree growth response along an elevational gradient: climate or genetics? Oecologia 173: 1587–1600. 23771802 10.1007/s00442-013-2696-6

[nph15348-bib-0049] Kölling C . 2007 Klimahüllen von 27 Waldbaumarten. AFZ‐DerWald 62: 1242–1245.

[nph15348-bib-0050] Kölling C , Zimmermann L . 2007 Die Anfälligkeit der Wälder Deutschlands gegenüber dem Klimawandel. Gefahrstoffe‐Reinhaltung der Luft 67: 259–268.

[nph15348-bib-0051] Körner C . 2007a Climatic treelines: conventiones, global patterns, causes. Erdkunde 61: 316–324.

[nph15348-bib-0052] Körner C . 2007b The use of ‘altitude’ in ecological research. Trends in Ecology & Evolution 22: 569–574. 17988759 10.1016/j.tree.2007.09.006

[nph15348-bib-0053] Kučera J . 2010 EMS 51 SAP FLOW SYSTEM for large tree trunks. [WWW document] URL http://emsbrno.cz/r.axd/pdf_v_EMS51__userman_u_pdf.jpg?ver= [accessed 24 May 2011].

[nph15348-bib-0054] Larcher W . 2003 Physiological plant ecology, *4*^*th*^*edn* Berlin, Germany: Springer‐Verlag.

[nph15348-bib-0055] Leo M , Oberhuber W , Schuster R , Grams TE , Matyssek R , Wieser G . 2014 Evaluating the effect of plant water availability on inner alpine coniferous trees based on sap flow measurements. European Journal of Forest Research 133: 691–698.

[nph15348-bib-0056] Levanič T , Gričar J , Gagen M , Jalkanen R , Loader NJ , McCarroll D , Oven P , Robertson I . 2009 The climate sensitivity of Norway spruce [*Picea abies* (L.) Karst.] in the southeastern European Alps. Trees – Structure and Function 23: 169–180.

[nph15348-bib-0057] Lévesque M , Rigling A , Bugmann H , Weber P , Brang P . 2016a Growth response of five co‐occurring conifers to drought across a wide climatic gradient in Central Europe. Agricultural and Forest Meteorology 197: 1–12.

[nph15348-bib-0058] Lévesque M , Saurer M , Siegwolf R , Eilmann B , Brang P , Bugmann H , Rigling A . 2013 Drought response of five conifer species under contrasting water availability suggests high vulnerability of Norway spruce and European larch. Global Change Biology 19: 3184–3199. 23712589 10.1111/gcb.12268

[nph15348-bib-0059] Lévesque M , Walthert L , Weber P . 2016b Soil nutrients influence growth response of temperate tree species to drought. Journal of Ecology. 104: 377–387.

[nph15348-bib-0060] Lindner M , Maroschek M , Netherer S , Kremer A , Barbati A , Garcia‐Gonzalo J , Seidl R , Delzon S , Corona P , Kolström M *et al* 2010 Climate change impacts, adaptive capacity, and vulnerability of European forest ecosystems. Forest Ecology and Management 259: 698–709.

[nph15348-bib-0061] Luis VC , Jiménez MS , Morales D , Kucera J , Wieser G . 2005 Canopy transpiration of a Canary Islands pine forest. Agricultural and Forest Meteorology 135: 117–123.

[nph15348-bib-0062] Matheny AM , Bohrer G , Garrity SR , Morin TH , Howard CJ , Vogel CS . 2015 Observations of stem water storage in trees of opposing hydraulic strategies. Ecosphere 6: 1–13.

[nph15348-bib-0063] Matyssek R . 1986 Carbon, water and nitrogen relations in evergreen and deciduous conifers. Tree Physiology 2: 177–187. 14975852 10.1093/treephys/2.1-2-3.177

[nph15348-bib-0064] Matyssek R , Wieser G , Patzner K , Blaschke H , Häberle K‐HH . 2009 Transpiration of forest trees and stands at different altitude: consistencies rather than contrasts? European Journal of Forest Research 128: 579–596.

[nph15348-bib-0065] Moser L , Fonti P , Büntgen U , Esper J , Luterbacher J , Franzen J , Frank D . 2010 Timing and duration of European larch growing season along altitudinal gradients in the Swiss Alps. Tree Physiology 30: 225–233. 20008326 10.1093/treephys/tpp108

[nph15348-bib-0066] Oberhuber W , Gruber A , Kofler W , Swidrak I . 2014 Radial stem growth in response to microclimate and soil moisture in a drought‐prone mixed coniferous forest at an inner Alpine site. European Journal of Forest Research 133: 467–479. 24883053 10.1007/s10342-013-0777-z PMC4035765

[nph15348-bib-0067] Oberhuber W , Hammerle A , Kofler W . 2015a Tree water status and growth of saplings and mature Norway spruce (*Picea abies*) at a dry distribution limit. Frontiers in Plant Science 6: 1–12. 26442019 10.3389/fpls.2015.00703 PMC4561357

[nph15348-bib-0068] Oberhuber W , Kofler W , Schuster R , Wieser G . 2015b Environmental effects on stem water deficit in co‐occurring conifers exposed to soil dryness. International Journal of Biometeorology. 59: 417–426. 24871430 10.1007/s00484-014-0853-1 PMC4346200

[nph15348-bib-0069] Oren R , Pataki DE . 2001 Transpiration in response to variation in microclimate and soil moisture in southeastern deciduous forests. Oecologia 127: 549–559. 28547493 10.1007/s004420000622

[nph15348-bib-0070] Oren R , Phillips N , Katul G , Ewers EB , Pataki ED . 1998 Scaling xylem sap flux and soil water balance and calculating variance: a method for partitioning water flux in forests. Annals of Forest Science 55: 191–216.

[nph15348-bib-0071] Pichler P , Oberhuber W . 2007 Radial growth response of coniferous forest trees in an inner Alpine environment to heat‐wave in 2003. Forest Ecology and Management 242: 688–699.

[nph15348-bib-0072] Pilcher JR . 1990 Sample preparation, cross‐dating and measurement In: Cook ER, Kairiukstis LA, eds. Methods of dendrochronology. Applications in the environmental sciences. Dordrecht, the Netherlands: Kluwer Academic Publishers, 40–51.

[nph15348-bib-0073] Pinheiro J , Bates D , DebRoy S , Sarkar D , R Core Team . 2017 nlme: Linear and Nonlinear Mixed Effects Models. [WWW document] URL https://cran.r-project.org/web/packages/nlme/index.html[accessedDecember2015].

[nph15348-bib-0074] R Core Team . 2016 R: a language and environment for statistical computing. Vienna, Austria: R Foundation for Statistical Computing.

[nph15348-bib-0075] Rebetez M , Reinhard M . 2007 Monthly air temperature trends in Switzerland 1901–2000 and 1975–2004. Theoretical and Applied Climatology 91: 27–34.

[nph15348-bib-0076] Rolland C , Petitcolas V , Michalet R . 1998 Changes in radial tree growth for *Picea abies, Larix decidua, Pinus cembra* and *Pinus uncinata* near the alpine timberline since 1750. Trees 13: 40–53.

[nph15348-bib-0077] Saulnier M , Roques A , Guibal F , Rozenberg P , Saracco G , Corona C , Edouard J‐L . 2017 Spatiotemporal heterogeneity of larch budmoth outbreaks in the French Alps over the last 500 years. Canadian Journal of Forest Research 47: 667–680.

[nph15348-bib-0078] Schmidt O . 2009 Fichtenwälder im klimawandel. Freising, Germany: Bayerische Landesanstalt für Wald und Forstwirtschaft.

[nph15348-bib-0079] Schulze E‐D , Cermák J , Matyssek R , Penka M , Zimmermann R , Vasicek F , Gries W , Kucera J , Čermák J , Matyssek M *et al* 1985 Canopy transpiration and water fluxes in the xylem of the trunk of *Larix* and *Picea* trees – a comparison of xylem flow, porometer and cuvette measurements. Oecologia 66: 475–483. 28310786 10.1007/BF00379337

[nph15348-bib-0080] Schuster R , Oberhuber W . 2013a Drought sensitivity of three co‐occurring conifers within a dry inner Alpine environment. Trees 27: 61–69. 23976821 10.1007/s00468-012-0768-6 PMC3750198

[nph15348-bib-0081] Schuster R , Oberhuber W . 2013b Age‐dependent climate‐growth relationships and regeneration of *Picea abies* in a drought‐prone mixed coniferous forest in the Alps. Canadian Journal of Forest Research 43: 609–618. 24027351 10.1139/cjfr-2012-0426 PMC3766819

[nph15348-bib-0082] Speer J, ed. 2010 Fundamentals of tree‐ring research. Tucson, AZ, USA: University of Arizona Press.

[nph15348-bib-0083] Staffler H , Karrer G . 2001 Wärmeliebende Wälder im Vinschgau (Südtirol/Italien). Sauteria 11: 301–358.

[nph15348-bib-0084] Staffler H , Karrer G . 2005 Die Schwarzföhrenforste im Vinschgau (Südtirol/Italien). Gredleriana 5: 135–170.

[nph15348-bib-0085] Stekhoven DJ , Bühlmann P . 2012 Missforest‐non‐parametric missing value imputation for mixed‐type data. Bioinformatics 28: 112–118. 22039212 10.1093/bioinformatics/btr597

[nph15348-bib-0086] Steppe K , De Pauw DJW , Lemeur R , Vanrolleghem PA . 2006 A mathematical model linking tree sap flow dynamics to daily stem diameter fluctuations and radial stem growth. Tree Physiology 26: 257–273. 16356899 10.1093/treephys/26.3.257

[nph15348-bib-0087] Swidrak I , Schuster R , Oberhuber W . 2013 Comparing growth phenology of co‐occurring deciduous and evergreen conifers exposed to drought. Flora – Morphology, Distribution, Functional Ecology of Plants 208: 609–617. 10.1016/j.flora.2013.09.004 PMC3836407 24273375

[nph15348-bib-0088] Tasser E , Leitinger G , Tappeiner U . 2017 Climate change versus land‐use change – what affects the mountain landscapes more? Land Use Policy 60: 60–72.

[nph15348-bib-0089] Thabeet A , Vennetier M , Gadbin‐Henry C , Denelle N , Roux M , Caraglio Y , Vila B . 2009 Response of *Pinus sylvestris* L. to recent climatic events in the French Mediterranean region. Trees 23: 843–853.

[nph15348-bib-0090] Thornthwaite CW , Mather J . 1951 The role of evapotranspiration in climate. Archiv für Meteorologie, Geophysik und Bioklimatologie, Serie B 3: 16–39.

[nph15348-bib-0091] Vacik H , de Jel S , Rubrecht H , Gruber G , Pividori M , Del Favero R , Klosterhuber R , Hotter M , Plettenbacher T , Aschaber R *et al* 2010a Waldtypisierung Südtirol Band 2 Waldgruppen, Naturräume, Glossar. Lana, Italy: Autonome Provinz Bozen‐Südtirol, Abteilung Forstwirtschaft, Amt für Forstplanung.

[nph15348-bib-0092] Vacik H , de Jel S , Rubrecht H , Gruber G , Pividori M , Del Favero R , Klosterhuber R , Hotter M , Plettenbacher T , Aschaber R *et al* 2010b Waldtypisierung südtirol band 1 waldtypen, wuchsgebiete, bestimmungsschlüssel. Lana, Italy: Autonome Provinz Bozen‐Südtirol, Abteilung Forstwirtschaft, Amt für Forstplanung.

[nph15348-bib-0093] Valentini R , Anfodillo T , Ehleringer JR . 1994 Water sources and carbon isotope composition (d^13^C) of selected tree species of the Italian Alps. Canadian Journal of Forest Research 24: 1575–1578.

[nph15348-bib-0094] Weber UM . 1997 Dendroecological reconstruction and interpretation of larch budmoth (*Zeiraphera diniana*) outbreaks in two central alpine valleys of Switzerland from 1470–1990. Trees 11: 277–290.

[nph15348-bib-0095] Wickham H . 2009 ggplot2: elegant graphics for data analysis. New York, NY, USA: Springer‐Verlag.

[nph15348-bib-0096] Wickham H . 2011 The split‐apply‐combine strategy for data analysis. Journal Of Statistical Software 40: 1–29.

[nph15348-bib-0097] Wieser G , Leo M . 2012 Whole‐tree water use by *Pinus cembra* at the treeline in the Central Tyrolean Alps. Plant Ecology & Diversity 5: 81–88.

[nph15348-bib-0098] Wolfslehner G , Koeck R , Hochbichler E , Steiner H , Frank G , Formayer H , Arbeiter F . 2011 Ökologische und waldbauliche eigenschaften der lärche (Larix decidua MILL.) – folgerungen für die waldbewirtschaftung in österreich unter berücksichtigung des klimawandels. Endbericht von Start‐Clim 2010 E in StartClim 2010: Anpassung an den Klimawandel: Weitere Beiträge zur Erstellung einer Anpassungsstrategie für Österreich Auftraggeber: BMLFUW, BMWF, BMWFJ, ÖBF.

[nph15348-bib-0099] Wood SN . 2006 Generalized additive models: an introduction with R. Boca Raton, FL, USA: Chapman and Hall/CRC.

[nph15348-bib-0100] Zang C , Biondi F . 2015 treeclim: an R package for the numerical calibration of proxy‐climate relationships. Ecography 38: 431–436.

[nph15348-bib-0101] Zeide B . 1993 Analysis of growth equations. Forest Science 39: 594–616.

[nph15348-bib-0102] Zeileis A , Grothendieck G . 2005 zoo: S3 infrastructure for regular and irregular time series. Journal Of Statistical Software 14: 1–27.

[nph15348-bib-0103] Zimmermann NE , Normand S , Pearman PB , Psomas A . 2013 Future ranges in European tree species In: Fitzgerald J, Lindner M, eds. Adapting to climate change in European forests – results of the MOTIVE project. Sofia, Bulgaria: Pensoft Publishers, 15–21.

[nph15348-bib-0104] Zimmermann R , Schulze E‐D , Wirth C , Schulze E‐E , McDonald KC , Vygodskaya NN , Waldemar Z . 2000 Canopy transpiration in a chronosequence of Central Siberian pine forests. Global Change Biology 6: 25–37.

[nph15348-bib-0105] Zuur AF , Ieno EN , Walker NJ , Saveliev AA , Smith GM . 2009 Mixed effects models and extensions in ecology with R. New York, NY, USA: Springer.

[nph15348-bib-0106] Zweifel R . 2016 Radial stem variations – a source of tree physiological information not fully exploited yet. Plant, Cell & Environment 39: 231–232. 10.1111/pce.12613 26184923

[nph15348-bib-0107] Zweifel R , Haeni M , Buchmann N , Eugster W . 2016 Are trees able to grow in periods of stem shrinkage? New Phytologist 211: 839–849. 27189708 10.1111/nph.13995

[nph15348-bib-0108] Zweifel R , Hasler R . 2001 Dynamics of water storage in mature subalpine *Picea abies*: temporal and spatial patterns of change in stem radius. Tree Physiology 21: 561–569. 11390300 10.1093/treephys/21.9.561

[nph15348-bib-0109] Zweifel R , Rigling A , Dobbertin M . 2009 Species‐specific stomatal response of trees to drought – a link to vegetation dynamics? Journal of Vegetation Science 20: 442–454.

[nph15348-bib-0110] Zweifel R , Zimmermann L , Newbery DM . 2005 Modeling tree water deficit from microclimate: an approach to quantifying drought stress. Tree Physiology 25: 147–156. 15574396 10.1093/treephys/25.2.147

[nph15348-bib-0111] Zweifel R , Zimmermann L , Zeugin F , Newbery DM . 2006 Intra‐annual radial growth and water relations of trees: implications towards a growth mechanism. Journal of Experimental Botany 57: 1445–1459. 16556628 10.1093/jxb/erj125

