# Supplementary material for: Water stress limits transpiration and growth of European larch up to the lower subalpine belt in an inner‐alpine dry valley
Source: New Phytol. 2018 Jul 20;220(2):460–75. doi: 10.1111/nph.15348 (PMC6586014; doi:10.1111/nph.15348)

**Water stress limits transpiration and growth of European larch up to the lower subalpine belt in an inner-alpine dry valley**

Nikolaus Obojes, Armin Meurer, Christian Newesely, Erich Tasser, Walter Oberhuber, Stefan Mayr, Ulrike Tappeiner.

Accepted: 2 June 2018

**Table S1a** Precipitation and snow cover for the three winter seasons (November until March) preceding the sap flow/dendrometer measuring seasons.

| Site | Year | Precipitation (mm) | Date of snow melt (DOY) | snow cover duration (d) | snow depth (cm) |
|---|---|---|---|---|---|
| S1200 | 2011/2012 | 99 | 2012-02-17 (48) | 32.5 | 6 |
| | 2012/2013 | 156[a] | 2012-12-26 (360) | 19 | 6 |
| | 2013/2014 | 232 | 2014-03-02 (61) | 65.5 | 16 |
| S1700 | 2011/2012 | 109 | 2012-03-09 (69) | 82.5 | 23 |
| | 2012/2013 | 153[a] | 2013-02-24 (55) | 89 | 26 |
| | 2013/2014 | 230 | 2014-03-24 (83) | 105 | 30 |
| S2000 | 2011/2012 | 115 | 2012-03-24 (84) | 110 | 37 |
| | 2012/2013 | 168[a] | 2013-04-17 (107) | 141 | 39 |
| | 2013/2014 | 238 | 2014-04-05 (95) | 134 | 34 |

[b] about half of total precipitation or more (94 mm at SF1, 75 mm at SF2, and 80 mm at SF3) in November 2012 (as rain not snow)

**Table S1b** Winter precipitation (November to March) for 2011/2012, 2012/2013, and 2013/2014 as well as long-term (1980-2010) averages and standard deviation at the climate station Marienberg/Monte Maria.

| Year | Precipitation (mm) |
|---|---|
| 2011/2012 | 91 |
| 2012/2013 | 215 [b] |
| 2013/2014 | 284 |
| 1981-2010 (ave. ± s.d.) | 188 ± 70 |

[b] more than half of total precipitation (118 of 215 mm) in November 2012 (as rain not snow)

**Table S2** Results of generalized additive mixed models to relate sap flow, daily radius change (DRC), and tree water deficits (TWD) to vapor pressure deficit (VPD), global radiation ($r_S$), precipitation (P), and soil water content (SWC). DOY indicates the time period, which was included in the model, N the number of observations (measuring days), adj. r² the variance explained, phi is a measure of autocorrelation. The estimate is given for the intercept, the slope for linear relations (linear-estimate), and the estimated degrees of freedom for non-linear smoothers (describing the shape of the smoother, smoother-edf). "_lag1" following the climate variables indicates when using a one day lag-period provided better results.

| | Sap flow | | | DRC | | | | | | TWD | | | | | |
|---|---|---|---|---|---|---|---|---|---|---|---|---|---|---|---|
| DOY | 150-250 (2013+2014 only) | | | Growth period (see Table 2) | | | 225-275 | | | Growth period (see Table 2) | | | 225-275 | | |
| N | | 561 | | | 279 | | | 457 | | | 279 | | | 459 | |
| adj. $R^2$ | | 0.88 | | | 0.19 | | | 0.63 | | | 0.30 | | | 0.71 | |
| phi | | 0.63 | | | 0.70 | | | 0.54 | | | 0.83 | | | 0.97 | |
| | linear-estimate | smoother - edf | Sig. p-value | linear-estimate | smoother - edf | linear-estimate | linear-estimate | smoother - edf | Sig. p-value | linear-estimate | smoother - edf | Sig. p-value | linear-estimate | smoother - edf | Sig. p-value |
| Intercept | 38.35 | | < 0.001 | 11.73 | | < 0.001 | | | n.s. | 3.23 | | n.s | 64.77 | | < 0.001 |
| VPD | | | | -1.45 | | < 0.01 | | 3.51 | < 0.001 | 1.67 | | < 0.001 | | | |
| VPD_lag1 | | | | | | | | | | | | | 3.58 | | < 0.001 |
| s(VPD):S1200 | | 3.90 | < 0.001 | | | | | | | | | | | | |
| s(VPD): S1700 | | 6.62 | < 0.001 | | | | | | | | | | | | |
| s(VPD):S2000 | | 1.44 | < 0.01 | | | | | | | | | | | | |
| $r_S$ | | | | | 1.66 | < 0.01 | | 3.55 | < 0.001 | 0.32 | | < 0.001 | | 4.57 | < 0.001 |
| s($r_S$): S1200 | | 5.18 | < 0.001 | | | | | | | | | | | | |
| s($r_S$): S1700 | | 4.88 | < 0.001 | | | | | | | | | | | | |
| s($r_S$): S2000 | | 4.44 | < 0.001 | | | | | | | | | | | | |
| P_lag1 | | | | | 3.72 | < 0.001 | | | | | 3.31 | < 0.001 | -0.56 | | < 0.001 |
| s(P_lag1): S1200 | | | | | | | | 1.67 | < 0.001 | | | | | | |
| s(P_lag1): S1700 | | | | | | | | 2.12 | < 0.001 | | | | | | |
| s(P_lag1): S2000 | | | | | | | | 1.50 | < 0.001 | | | | | | |
| s(SWC_lag1): S1200 | | 4.95 | < 0.001 | | | | | | | | | | | 3.68 | < 0.001 |
| s(SWC_lag1): S1700 | | 5.71 | < 0.001 | | | | | | | | | | | 5.24 | < 0.001 |
| s(SWC_lag1): S2000 | | 6.6E-07 | n.s. | | | | | | | | | | | 0.00 | n.s. |

**Table S3** Growth reactions of trees to selected pointer years. The percentage of trees per site showing at least a 25% increase (+) or 25 % decrease (-) in total tree ring width compared to the previous year is displayed (light and dark green colors mark years with a positive growth reaction of 50-75% and 76-100% of trees per site, respectively; orange and red colors mark years with a negative growth reaction of 50-75% and 76-100% of trees per site, respectively). Dry and/or hot years (1976, 2003, 2004) led to negative growth reaction at low elevation, while trees at the forest line might even react positively (1976). Wet and cold years (1975, 1977) led to positive reactions at low elevation and negative ones at high elevation.

| | | Weather conditions / Percentage of trees per site showing a growth reaction | | | | | | | | |
|---|---|---|---|---|---|---|---|---|---|---|
| | | 1947 | 1948 | 1949 | 1975 | 1976 | 1977 | 2002 | 2003 | 2004 |
| Site | Growth reaction | warm, humid 1st - dry 2nd half | warm until Aug, humid | cold, dry summer | humid, cold summer | winter to summer dry and hot, wet autumn | cold, wet summer | dry and cold summer | dry and hot spring and summer | rather cool, dry |
| S1100 | + | 86 | 0 | 14 | 100 | 0 | 100 | 100 | 0 | 0 |
| | - | 0 | 100 | 86 | 0 | 100 | 0 | 0 | 71 | 100 |
| S1200 | + | | | | 93 | 0 | 93 | 0 | 0 | 0 |
| | - | | | | 0 | 100 | 0 | 20 | 67 | 100 |
| S1700 | + | 20 | 13 | 0 | 93 | 7 | 40 | 13 | 0 | 0 |
| | - | 0 | 0 | 47 | 0 | 73 | 7 | 0 | 67 | 100 |
| S2000 | + | 11 | 10 | 60 | 15 | 31 | 31 | 0 | 15 | 0 |
| | - | 67 | 20 | 0 | 23 | 0 | 0 | 8 | 8 | 31 |
| S2200 | + | | | | 0 | 100 | 0 | 0 | 29 | 0 |
| | - | | | | 100 | 0 | 43 | 14 | 0 | 14 |

**Fig. S1** Regression lines and smoothers for the general additive mixed models between sap flow (**a-b**), daily radius change (DRC, **c-d**) as well as tree water deficits (TWD, **e-f**) and potential evaporation (PET) as well as water availability expressed by precipitation (P) and/or soil water content (SWC). The sap flow model was calculated for the whole summer (DOY 150-250), the DRC- and TWD-model for the growth period as defined in Table 2. Lines in color display site-specific relations (i.e. the relation between sap flow and the explanatory variable differs between sites), black lines non-site-specific ones. For linear relations the estimate given in table 3 describes the slope of the line. For non-linear relations the parameter edf given in table 3 characterizes the shape of the smoother curve, the higher the value, the more non-linear the relation. "_lag1" following the climate variables indicates when using a one day lag-period provided better results.

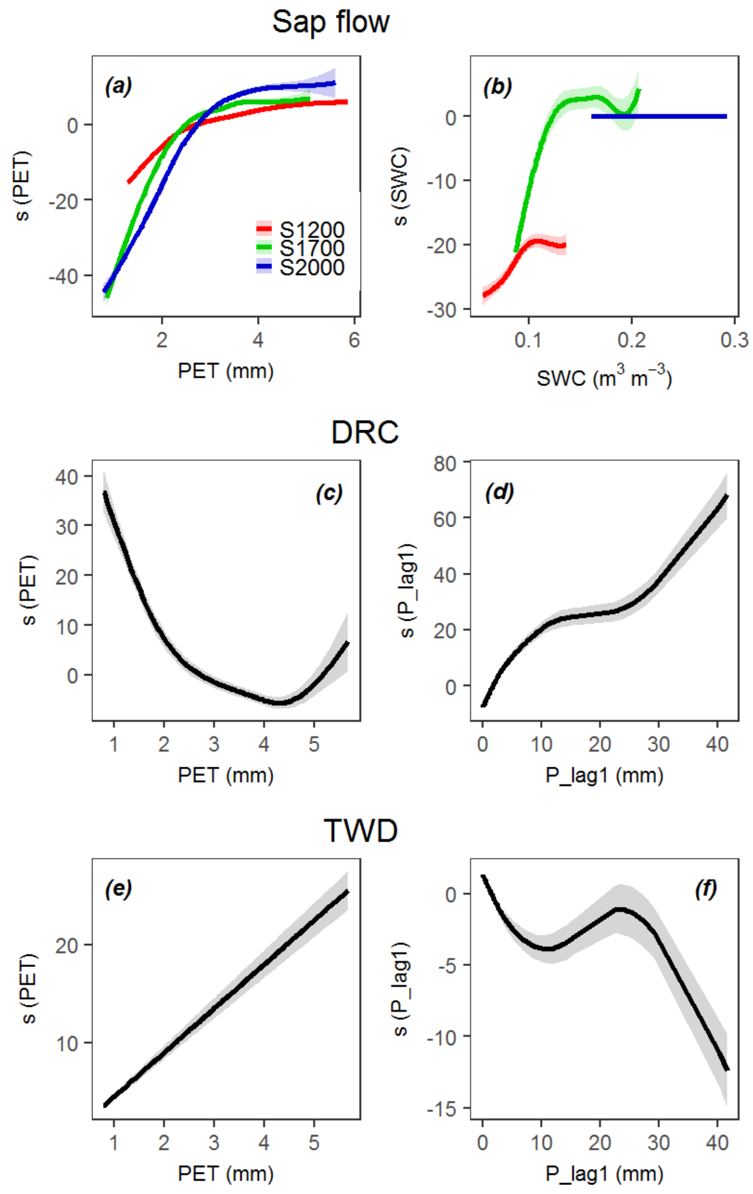

**Fig. S2** Regression lines and smoothers for the late summer period (DOY 225-275) for the general additive mixed models between daily radius change (DRC, **a**-**b**) as well as tree water deficits (TWD, **c**-**e**) and potential evaporation (PET) as well as water availability expressed by precipitation (P) and/or soil water content (SWC). Lines in color display site-specific relations, black lines non-site-specific ones. For linear relations the estimate given in table 3 describes the slope of the line. For non-linear relations the parameter edf given in table 3 characterizes the shape of the curve, the higher the value, the more non-linear the relation. The horizontal line for the TWD-SWC plot for S2000 indicates a non-significant relation. "_lag1" following the climate variables indicates when using a one day lag-period provided better results, "P last 3" is the sum of precipitation of the current and the two previous days.

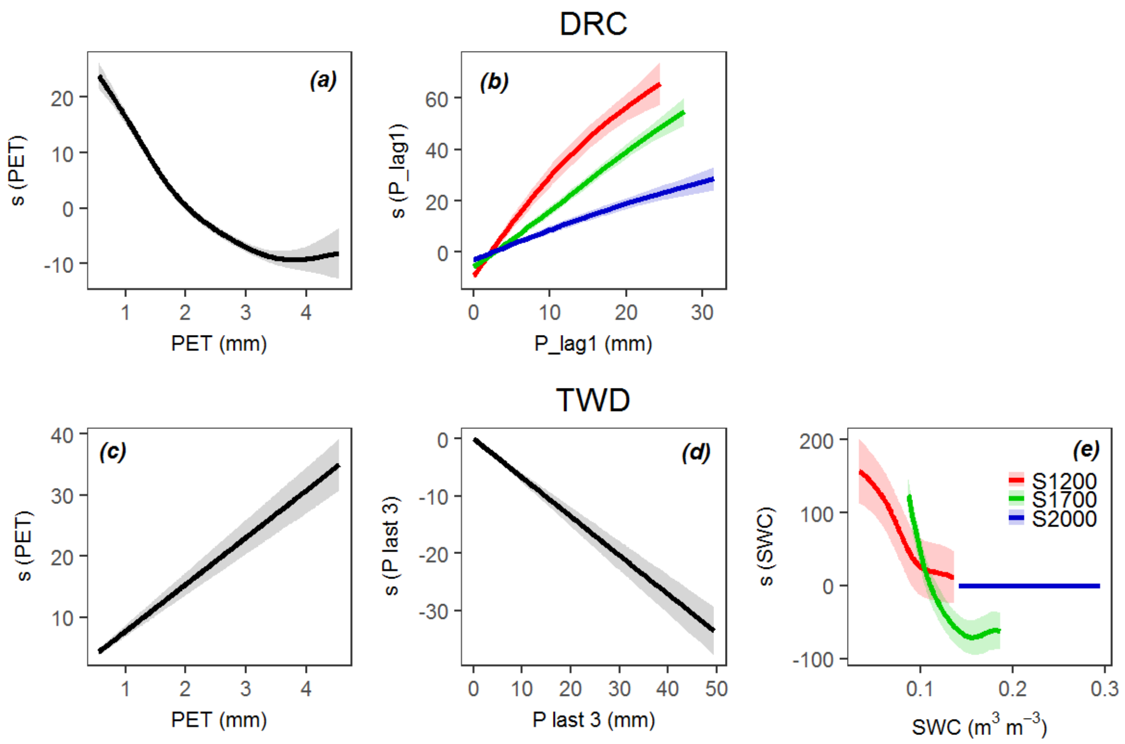

**Fig. S3** Regression lines and smoothers for the general additive mixed models between sap flow (**a-c**), daily radius change (DRC, **d-f**) as well as tree water deficits (TWD, **g-i**) and vapor pressure deficit (VPD), global radiation ($r_S$), precipitation (P), and soil water content (SWC). The sap flow model was calculated for the whole summer (DOY 150-250) and the DRC- and TWD-model for the growth period as defined in Table 2. Lines in color display site-specific relations (i.e. the relation between sap flow and the explanatory variable differs between sites), black lines non-site-specific ones. For linear relations the estimate given in table 3 describes the slope of the line. For non-linear relations the parameter edf given in table 3 characterizes the shape of the curve, the higher the value, the more non-linear the relation. The horizontal line for the sap flow-SWC plot for S2000 indicates a non-significant relation. "_lag1" following the climate variables indicates when using a one day lag-period provided better results.

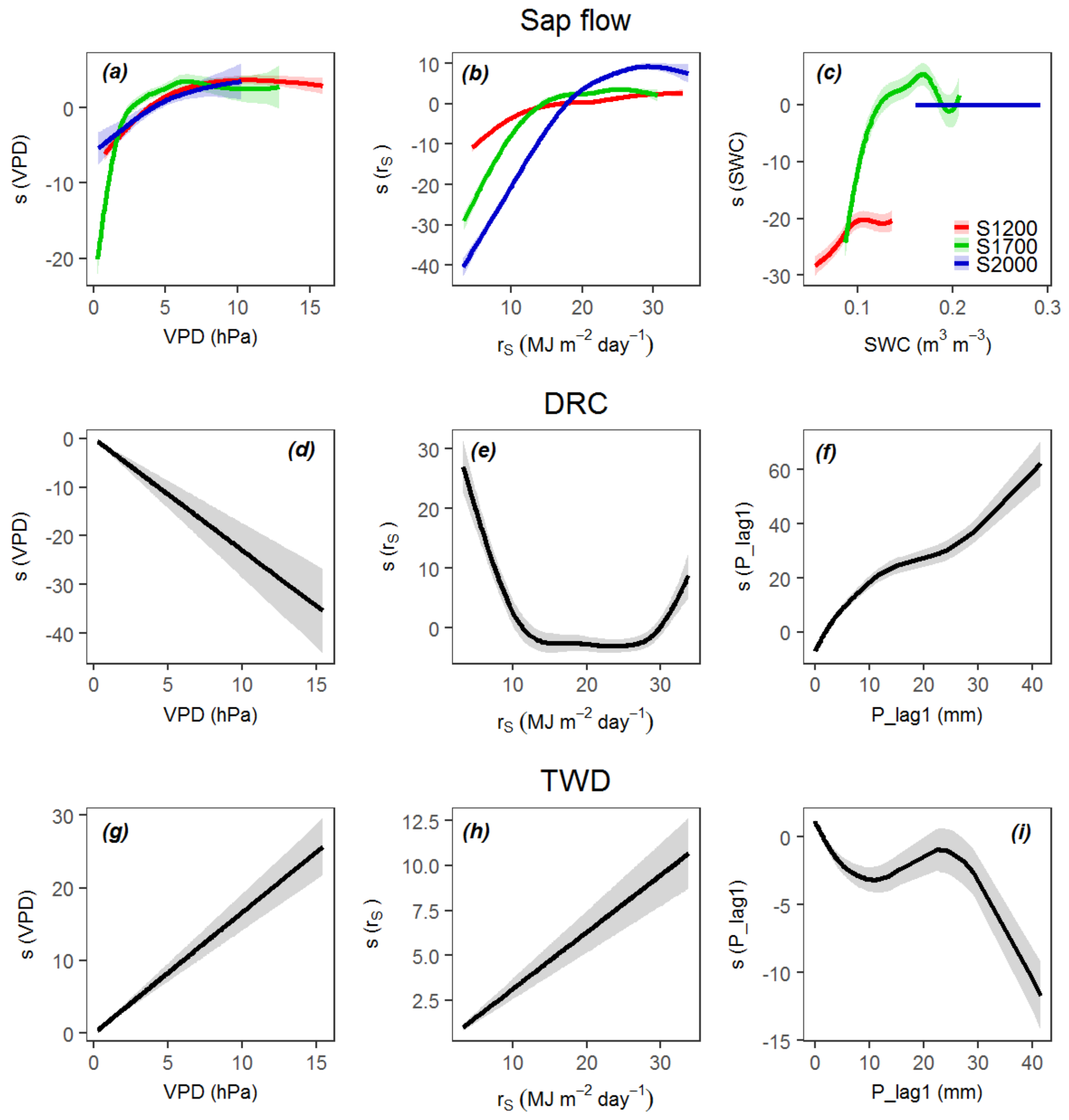

**Fig. S4** Regression lines and smoothers for the general additive mixed models between daily radius change (DRC, **a-c**) as well as tree water deficits (TWD, **d-g**) and vapor pressure deficit (VPD), global radiation ($r_S$), precipitation (P), and soil water content (SWC) for the late summer period (DOY 225-275). Lines in color display site-specific relations, black lines non-site-specific ones. For linear relations the estimate given in table 3 describes the slope of the line. For non-linear relations the parameter edf given in table 3 characterizes the shape of the curve, the higher the value, the more non-linear the relation. The horizontal line for the TWD-SWC plot for S2000 indicates a non-significant relation. "_lag1" following the climate variables indicates when using a one day lag-period provided better results.

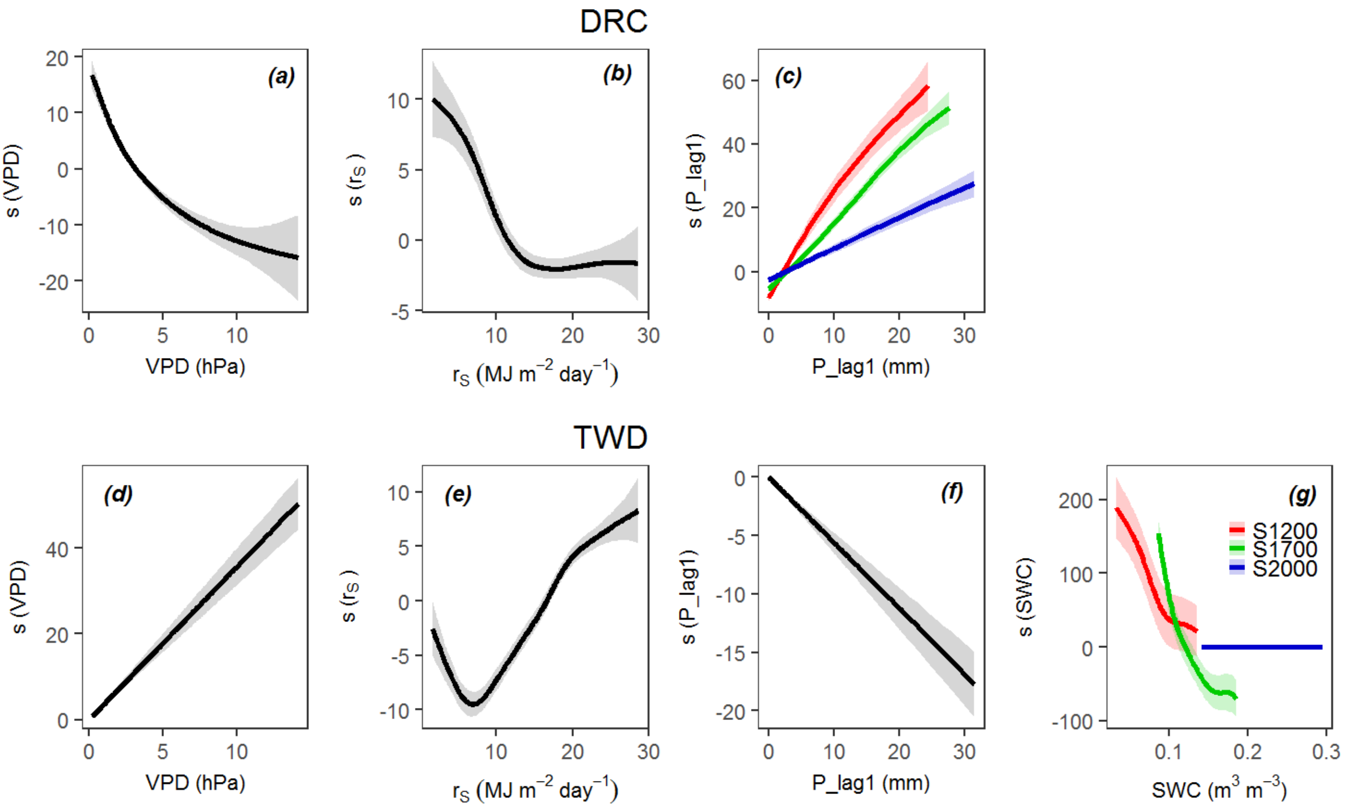

**Fig. S5** Static response function analysis between residual chronologies and monthly mean temperature (T) and total precipitation (P) for periods with a sampling depth of at least five trees per site. Full circles indicate significant relationships at $p < 0.1$. Months in lowercase denote the previous, in capital letters the actual year of the tree ring formation.
There is positive growth response of lower elevation sites (up to S1700) to spring precipitation, S1100 and S1200 also react positively to previous autumn and January precipitation. Temperature responses are positive at the forest line in July which is probably the main growth period there, in May at S1700 (and slightly not significant at S2000) when cold temperatures might delay growth at these elevations and in previous autumn for S2200 and S1700 (and slightly not significant at S2000). The negative temperature response in April at S2200 and S2000 is likely linked to frost dryness in early spring; in January at S1100 it could also be due to precipitation falling as rain instead of snow which means less water early in the growing season. A negative response to previous summer T (at S2000 in July) is reported in literature.

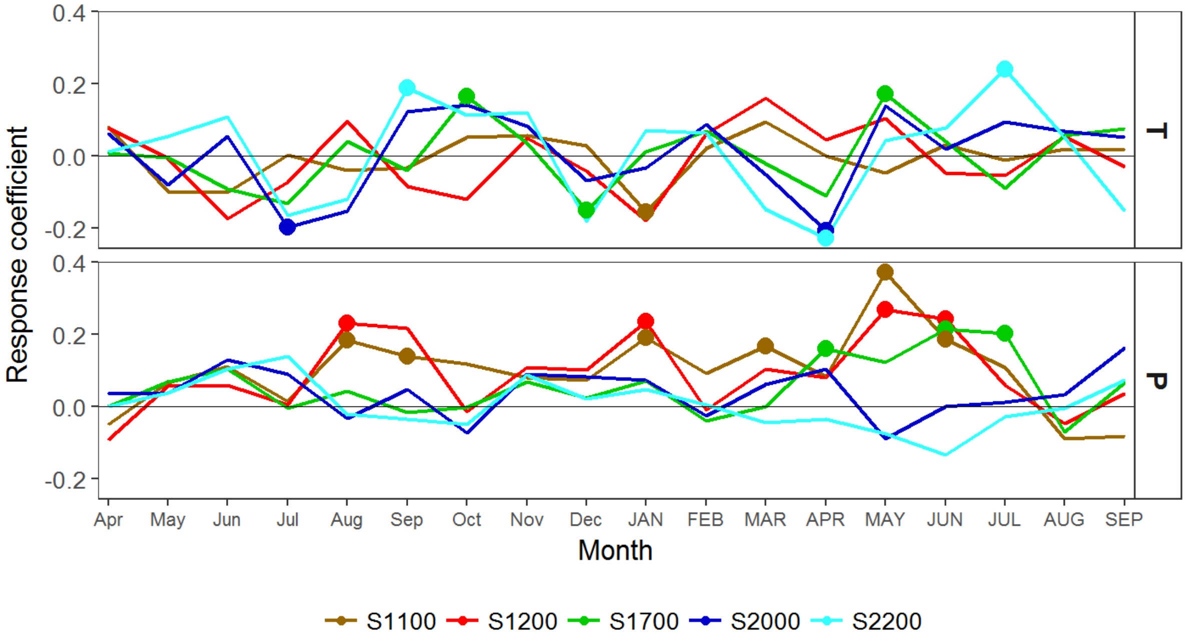

**Fig. S6** Moving 25-year response function analysis between residual chronologies and monthly mean temperature (T) and total precipitation (P) for periods with a sampling depth of at least five trees per site for selected months in the previous (lower case) and current (capital letters) year (the months not shown in Fig. 5). Full circles indicate significant relationships at $p < 0.1$. Generally precipitation responses peaked in recent decades (also see Fig. 5 in the main text) while most temperature responses were most significant for 25-year periods ending in the 1980s and 1990s.

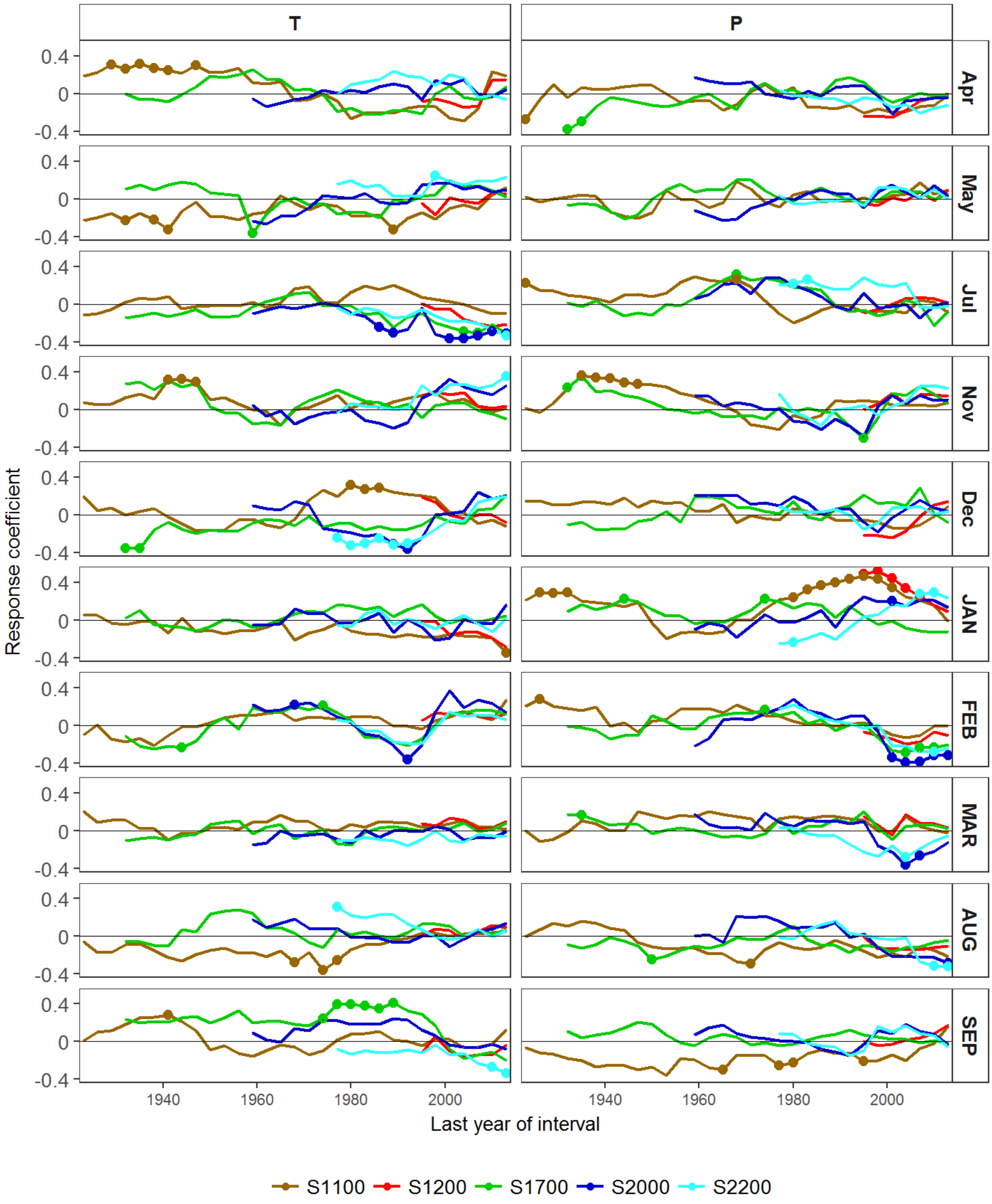

Supplement: Supplementary file 1 — Fig. S1 Regression lines and smoothers for the general additive mixed models between sap flow, daily radius change as well as tree water deficits and potential evaporation as well as water availability expressed by precipitation and/or soil water content. Fig. S2 Regression lines and smoothers for the late summer period (DOY 225–275) for the general additive mixed models between daily radius change as well as tree water deficits and potential evaporation as well as water availability expressed by precipitation and/or soil water content. Fig. S3 Regression lines and smoothers for the general additive mixed models between sap flow, daily radius change as well as tree water deficits and vapor pressure deficit, global radiation, precipitation, and soil water content. Fig. S4 Regression lines and smoothers for the general additive mixed models between daily radius change as well as tree water deficits and vapor pressure deficit, global radiation, precipitation, and soil water content for the late summer period (DOY 225–275). Fig. S5 Static response function analysis between residual chronologies and monthly mean temperature and total precipitation. Fig. S6 Moving 25 yr response function analysis between residual chronologies and monthly mean temperature and total precipitation. Table S1 Precipitation and snow cover for the three winter seasons preceding the sap flow/dendrometer measuring seasons as well as long‐term (1980–2010) averages; winter precipitation (November to March) for 2011/2012, 2012/2013, and 2013/2014 as well as long‐term (1980–2010) averages and standard deviation at the climate station Marienberg/Monte Maria Table S2 Results of generalized additive mixed models to relate sap flow, daily radius change, and tree water deficits to vapor pressure deficit, global radiation, precipitation, and soil water content Table S3 Growth reactions of trees to selected pointer years [file NPH-220-460-s001.pdf]
